# Active Tumor-Targeting Nano-formulations Containing Simvastatin and Doxorubicin Inhibit Melanoma Growth and Angiogenesis

**DOI:** 10.3389/fphar.2022.870347

**Published:** 2022-04-05

**Authors:** Giorgiana Negrea, Valentin-Florian Rauca, Marta Szilvia Meszaros, Laura Patras, Lavinia Luput, Emilia Licarete, Vlad-Alexandru Toma, Alina Porfire, Dana Muntean, Alina Sesarman, Manuela Banciu

**Affiliations:** ^1^ Doctoral School in Integrative Biology, Faculty of Biology and Geology, “Babes-Bolyai” University, Cluj-Napoca, Romania; ^2^ Department of Molecular Biology and Biotechnology, Center of Systems Biology, Biodiversity and Bioresources, Faculty of Biology and Geology, “Babes-Bolyai” University, Cluj-Napoca, Romania; ^3^ Department of Dermatology and Allergology, School of Medicine, Technical University of Munich, Munich, Germany; ^4^ Molecular Biology Centre, Institute for Interdisciplinary Research in Bio-Nano-Sciences, Babes-Bolyai University, Cluj-Napoca, Romania; ^5^ Department of Experimental Biology and Biochemistry, Institute of Biological Research, Branch of NIRDBS Bucharest, Cluj-Napoca, Romania; ^6^ Department of Pharmaceutical Technology and Biopharmaceutics, Faculty of Pharmacy, University of Medicine and Pharmacy “Iuliu Hatieganu”, Cluj-Napoca, Romania

**Keywords:** melanoma, drug delivery systems, liposomes, extracellular vesicles, simvastatin, doxorubicin, tumor microenvironment

## Abstract

Primary melanoma aggressiveness is determined by rapid selection and growth of cellular clones resistant to conventional treatments, resulting in metastasis and recurrence. In addition, a reprogrammed tumor-immune microenvironment supports melanoma progression and response to therapy. There is an urgent need to develop selective and specific drug delivery strategies for modulating the interaction between cancer cells and immune cells within the tumor microenvironment. This study proposes a novel combination therapy consisting of sequential administration of simvastatin incorporated in IL-13-functionalized long-circulating liposomes (IL-13-LCL-SIM) and doxorubicin encapsulated into PEG-coated extracellular vesicles (PEG-EV-DOX) to selectively target both tumor-associated macrophages and melanoma cells. To this end, IL-13 was conjugated to LCL-SIM which was obtained *via* the lipid film hydration method. EVs enriched from melanoma cells were passively loaded with doxorubicin. The cellular uptake of rhodamine-tagged nano-particles and the antiproliferative potential of the treatments by using the ELISA BrdU-colorimetric immunoassay were investigated *in vitro*. Subsequently, the therapeutic agents were administered *i.v* in B16.F10 melanoma-bearing mice, and tumor size was monitored during treatment. The molecular mechanisms of antitumor activity were investigated using angiogenic and inflammatory protein arrays and western blot analysis of invasion (HIF-1) and apoptosis markers (Bcl-xL and Bax). Quantification of oxidative stress marker malondialdehyde (MDA) was determined by HPLC. Immunohistochemical staining of angiogenic markers CD31 and VEGF and of pan-macrophage marker F4/80 was performed to validate our findings. The *in vitro* data showed that IL-13-functionalized LCL were preferentially taken up by tumor-associated macrophages and indicated that sequential administration of IL-13-LCL-SIM and PEG-EV-DOX had the strongest antiproliferative effect on tumor cells co-cultured with tumor-associated macrophages (TAMs). Accordingly, strong inhibition of tumor growth in the group treated with the sequential combination therapy was reported *in vivo*. Our data suggested that the antitumor action of the combined treatment was exerted through strong inhibition of several pro-angiogenic factors (VEGF, bFGF, and CD31) and oxidative stress-induced upregulation of pro-apoptotic protein Bax. This novel drug delivery strategy based on combined active targeting of both cancer cells and immune cells was able to induce a potent antitumor effect by disruption of the reciprocal interactions between TAMs and melanoma cells.

## Introduction

Melanoma incidence has been rising worldwide, far outpacing the efforts to counteract the aggressiveness of the highly heterogeneous disease (Beiu et al., 2020; Tasdogan et al., 2020). In this regard, the involvement of different constituents of the TME in melanoma development needs to be better understood in order to elucidate the mechanisms of environment-mediated tumor growth, invasion, angiogenesis, and drug resistance (Pautu et al., 2017). As primary melanoma is associated with Th2-mediated chronic inflammation and VEGF overexpression in favor of tumor progression and metastasis (Nevala et al., 2009; Grotz et al., 2015), targeting of both Th2 polarization and VEGF-mediated angiogenesis in early-stage melanoma may constitute a viable therapeutic option. IL-4 and IL-13 cytokines are the established inducers of Th2 response, and overexpression of their receptors on tumor cells can be targeted by cancer therapies (Suzuki et al., 2015). Among IL-13 receptors, the heterodimeric IL-13R*α*1 is shared by both IL-4 and IL-13 ligands, being expressed in tumors, as well as in normal tissues. In contrast, the monomeric IL-13R*α*2 receptor is mostly absent in normal tissues, it is overexpressed in melanoma and other solid tumors, and it constitutes a “private chain” for high-affinity binding and sequestration of IL-13, serving as a potential target for counteracting melanoma progression (Suzuki et al., 2015; Okamoto et al., 2019; Gupta et al., 2021). The M2-like phenotype typical for TAMs is induced by Th2-derived cytokines among which IL-13 plays a central role (Sinha et al., 2005; Jayasingam et al., 2019); therefore, targeting IL-13 receptors/ligands in the tumor microenvironment can inhibit M2 polarization and ultimately reduce tumor burden (Okamoto et al., 2019; Duan and Luo, 2021).

Tumor cell-derived diffusible molecules and transport vesicles can recruit and polarize TAMs and other immune cells to promote pro-angiogenic signaling pathways *via* a wide range of molecules such as bFGF, VEGF, IL-1, IL-8, TNF-*α*, MMPs, and NO (Pan et al., 2020; SenGupta et al., 2021). Many of our previous studies highlighted the importance of targeting the tumor cell–TAMs axis in melanoma (Alupei et al., 2015; Rauca et al., 2018; Licarete et al., 2020; Rauca et al., 2021). The study by Alupei et al. (2015) demonstrated that the antitumor activity of liposomal simvastatin is elicited *via* modulation of TAM-mediated oxidative stress in the melanoma microenvironment. Using an *in vitro* melanoma model under hypoxia-mimicking conditions (Rauca et al., 2018), it was reported that simvastatin in combination with a vascular disrupting agent induced an unfavorable microenvironment for B16.F10 cell proliferation and migration by suppressing HIF-1*α* protein levels in tumor cells and downregulating the expression of Arg-1 in TAMs. Furthermore, to specifically target tumor cells, we developed a novel system based on B16.F10 melanoma cell-enriched EVs stabilized with PEG and loaded with DOX that elicited a higher antitumor efficacy than liposomal DOX *via* more efficient delivery of the therapeutic agent compared to commercially available nano-formulations (Doxil^®^) (Patras et al., 2021).

In this light, the aim of the present study was to develop an improved targeted therapy for melanoma using an optimized nano-system based on Il-13-conjugated long-circulating liposomes containing SIM (Il-13-LCL-SIM) to target TAMs and PEG-stabilized EVs containing DOX (PEG-EV-DOX) to target melanoma cells. A recent study by Haas et al. (2021) provided strong scientific rationale for dealing first with the immune-evasive TME before the settlement of resistance to the targeted therapy, to avoid cross resistance in melanoma patients. In accordance with these findings, in this study, we administered the drugs to B16.F10 melanoma models *in vitro* and *in vivo* in a sequential fashion. The Th2-mediated immune infiltration was targeted first by using IL-13-LCL-SIM. After 24 h, PEG-EV-DOX was used as “trojan horses” (Patras et al., 2021) to deliver DOX to tumors. Our results have shown that IL-13-conjugated liposomes were preferentially taken up by M2 macrophages and the sequential targeted therapy consisting of IL-13-LCL-SIM and PEG-EV-DOX was most effective in inhibiting B16.F10 melanoma growth *via* strong reduction of TME angiogenic capacity and macrophage infiltration, as well as by inducing an oxidative stress-related increase in tumor apoptotic status. This novel targeted therapy has the advantage of sequentially targeting major processes involved in melanoma development with higher efficiency, by using both the advantage of superior tumor targeting of LCL and EVs, as well as the higher affinity toward M2 macrophages given by IL-13 conjugation of the liposomal formulation.

## Materials and Methods

### Preparation of Nano-formulations

To prepare the liposomal formulations, DPPC and PEG-2000-DSPE were purchased from Lipoid GmbH (Ludwigshafen, GER), CHL and SIM were acquired from Sigma-Aldrich Chemie GmbH (Munich, GER), and DSPE-PEG-2000-MI and rhodamine B were acquired from Avanti Polar Lipids (Darmstadt, GER). LCL-SIM were prepared using the following molar ratio between lipids and the lipophilic statin: 14.984:3:0.279:0.731:1.209 (DPPC:Chol:PEG-2000-DSPE:DSPE-PEG-2000-MI:SIM), adapted from protocols in our previous publications (Banciu et al., 2008a; Alupei et al., 2015; Rauca et al., 2021). The starting concentration of DPPC was 20 mM. To confer fluorescent properties to the LCL formulations for the *in vitro* uptake studies, rhodamine B (0.1% from the total amount of phospholipids) was introduced in the mix, excepting the LCL formulations used to assess B16.F10 proliferation capacity. After dissolving the lipids in 100% ethanol in a rotary evaporator, the previously described lipid film hydration method (Alupei et al., 2015) was used to obtain LCL-SIM nano-formulations.

To obtain IL-13-LCL-SIM by IL-13 conjugation, we adapted a previously described protocol by Madhankumar et al. (2009). In brief, prior to conjugation, IL-13 was thiolated by incubation with immunothiolane hydrochloride (ratio 1:40) for 1 h at room temperature with gentle continuous shaking. The thiolation reaction was stopped by incubating the solution for 1 h with 20 mM *β*-mercaptoethanol and gentle shaking. To remove excess of immunothiolane and *β*-mercaptoethanol, thiolated IL-13 was washed four times in 3 kDa molecular weight cutoff tubes. Next, thiolated IL-13 was conjugated with LCL-SIM overnight at 4°C, at a ratio of 1:170 (IL13: DSPE-PEG-2000-MI). After conjugation, the maleimide in DSPE-PEG-2000-MI was inactivated by incubating the solution for 1 h with 20 mM *β*-mercaptoethanol and gentle shaking. To eliminate *β*-mercaptoethanol from liposome solution, the sample was dialyzed against PBS pH 7.2 overnight at 4°C. To remove aggregates, the liposome solution was centrifuged at 5,000 × g, for 1 h at 4°C. Following lipid removal with 100% ethanol, simvastatin concentration in LCL-SIM and IL-13-LCL-SIM was determined spectrophotometrically (239 nm) using 0.6–20 μg/ml SIM standards.

The complete isolation, purification, PEG functionalization, and physicochemical characterization steps for obtaining PEG-EV-DOX used in the present study were presented in detail in a recently published manuscript by our group (Patras et al., 2021).

### Cell Types and Culture Conditions

B16.F10 murine melanoma cells (ATCC and CRL-6475) were cultured in DMEM (Lonza, Basel, CH) as the monolayer in 5% CO_2_ humidified atmosphere as detailed in our previous article (Licarete et al., 2017). Procedures regarding obtaining BMDMs were carried out in strict accordance with the recommendations in the European (Directive 2010/63/EU) and national legislation (43/2014). The protocol was approved by the Committee on the Ethics of Animal Experiments of Babes-Bolyai University (registration no. 31444/27.03.2017). After euthanasia by CO_2_ inhalation, bone marrow progenitor cells were isolated by flushing the marrow from the femurs of 8-week-old male C57BL/6 mice (Cantacuzino Institute, Bucharest, RO). After isolation, bone marrow cells were cultured and differentiated in DMEM supplemented with 10 ng/ml M-CSF (Cell Signaling Technology, MA, United States) to obtain BMDMs, as previously described (Rauca et al., 2018). For the proliferation assay, melanoma cells were co-cultured with BMDMs at a cell density ratio of 1:4, which was shown to optimally mimic melanoma microenvironment conditions (Rauca et al., 2018).

### Quantification of Nanoparticle Uptake by M1/M2 Macrophages and B16 Melanoma Cells

Tumor cells (5 × 10^3^ cells/well) and BMDMs (2 × 10^4^ cells/well) were seeded separately in clear-bottom 96-well black plates. On day seven, BMDMs were incubated with either 20 ng/ml IL-4 (Cell Signaling Technology, MA, United States) or 20 ng/ml LPS (Sigma–Aldrich Chemie GmbH, Munich, Germany) to promote polarization into M2/M1 macrophages (Georgoudaki et al., 2016). On day eight, all cells were treated with empty liposomes (LCL without SIM) and empty liposomes conjugated with IL-13 (IL-13-LCL without SIM). Determination of phospholipid concentration was carried out by colorimetric determination (797 nm) of inorganic phosphate from lipids extracted in the organic phase, according to the work of Rouser et al. (1970), to ensure the administration of the same lipid concentration (3.3 mM) for uptake comparison. Each treatment was applied in triplicate. Untreated B16.F10 cells and M1 and M2 macrophages incubated with the culture medium were used to record cell autofluorescence. After 5, 15, and 30 min incubation with each liposomal formulation, the culture medium was removed and cells were washed three times with PBS. After washing, 100 µL PBS was added to the wells and fluorescence intensity, expressed as RFU, was measured using a FLUOstar Omega (BMG Labtech) plate reader. For rhodamine B, the fluorescent component of LCL formulations, the excitation wavelength was set at 540 nm and the emission wavelength at 580 nm.

### Cell Proliferation Assay

To assess the *in vitro* efficacy of our sequential treatment strategy, we first designed an experimental setup that allowed us to co-culture B16.F10 melanoma cells and M2 macrophages previously treated with LCL devoid of drug or with different concentrations of LCL-SIM and IL-13-LCL-SIM. For this setup, on day seven after differentiation, BMDMs were seeded into 96-well plates (8 × 10^3^ cells/well) and treated with 20 ng/ml IL-4 to undergo M2 polarization. On day eight, the M2 macrophages were treated with different concentrations of SIM encapsulated in LCL-SIM or IL-13-LCL-SIM. On day nine, the medium containing liposomal treatments was replaced with a fresh untreated medium containing 2 × 10^3^ B16.F10 cells. At the end of the day, BrDU was added to the cells overnight. The proliferative activity of B16.F10 melanoma cells in each experimental setup after different treatments was tested using ELISA BrdU-colorimetric immunoassay (Roche Applied Science, Penzberg, DE) as previously described (Licarete et al., 2017; Rauca et al., 2018). In a similar setup, the effect of different concentrations of PEG-EV-DOX was tested on B16.F10 cells co-cultured with untreated or IL-13-LCL-SIM-treated M2 macrophages. The IC_50_ values of PEG-EV-DOX treatment effect on B16.F10 cell proliferation in monoculture and in co-culture with untreated TAMs and IL-13-LCL-SIM-treated TAMs were calculated. Results were expressed as % of proliferation compared to control (Rauca et al., 2019) (untreated cells in monoculture and in co-culture, respectively).

### Murine Tumor Model

To induce melanoma tumors *in vivo*, 1 × 10^6^ B16.F10 cells were injected in the right-flank of 6–8-week-old male C57BL/6 mice (Cantacuzino Institute, Bucharest, RO), kept under standard laboratory conditions. During treatment, tumor size and body weight were monitored on a daily basis. The time points for the sequential administration of IL-13-LCL-SIM and PEG-EV-DOX were chosen based on the treatment scheme used in our recent study (Patras et al., 2021) in which we reported significant antitumor effects of PEG-EV-DOX, when injected in C57BL6 mice, on days 8 and 11. As our main goal was to optimize the therapeutic effects of PEG-EV-DOX by counteracting the tumor microenvironment-induced resistance, we administered IL-13-LCL-SIM one day in advance of PEG-EV-DOX, on days 7 and 10 (Supplementary Figure S1). The nano-formulations were injected intravenously (Rauca et al., 2021) as follows: 5 mg/kg IL-13-LCL-SIM on days 7 and 10; 2 mg/kg PEG-EV-DOX on days 8 and 11 (Supplementary Figure S1). Mice from the group treated with the combination of both received the nano-formulations sequentially on the aforementioned dates. Each experimental group consisted of five animals, and an untreated control group was used. At the end of the experiment (day 12), mice were euthanized by CO_2_ inhalation in euthanasia chambers, with minimum suffering. Animal experiments were performed according to the EU Directive 2010/63/EU and to the national regulations. The study was reported in accordance with ARRIVE guidelines and was approved by the Babes-Bolyai University Ethics Committee (Cluj-Napoca, Romania; Project ID: PN-III-P4-ID-PCE-2016-0342, contract no. 91/2017, within PNCDI III Approval no. 4335/19.03.2018).

### Treatment Effect on Tumor Growth

To investigate the efficacy of the new liposomal formulation encapsulating SIM, 5 mg/kg free SIM, LCL-SIM, and IL-13-LCL-SIM were administered *i.v*. on day 7 after tumor cell inoculation, when tumors reached an average size of 50 mm^3^, and re-administered on day 10. In parallel, as reported in our recently published study (Patras et al., 2021), 2 mg/kg DOX was administered in either free form or incorporated in PEG-EVs or encapsulated in LCL on days 8 and 11 after tumor cell inoculation. The combination therapy consisted of sequential administration of IL-13-LCL-SIM and PEG-EV-DOX on the same experimental group. The formula *V* = 0.52a^2^b, where “a” is the smallest and “b” is the largest superficial diameter (measured with calipers), was used to determine tumor volume (Licarete et al., 2020). Mice from all experimental groups were euthanized on day 12, and tumors were collected and stored in liquid nitrogen for further analysis.

### Tumor Tissue Lysate Preparation

At day 12, when the tumor size reached around 1,000 mm^3^, the mice were euthanized using CO_2_ anoxia and the tumors were isolated, weighed, instantly frozen in liquid nitrogen, and stored at −80°C. Later, the tumors were pooled (five tumors for each experimental group) and incubated for 30 min on ice in lysis buffer supplemented with protease and phosphatase inhibitors (Luput et al., 2018). Next, the lysate was mechanically homogenized using a Potter (Thomas Scientific, New Jersey, United States) and further centrifuged at 15,000 × g for 10 min at 4°C. The supernatant was collected, and the protein concentration of the pooled lysates in each experimental group was measured by the biuret method (Gornall et al., 1949). The tumor lysates were stored at −80°C and further used for various molecular investigations.

### Protein Array Profiling of Tumor Angiogenesis

To determine whether the tested nano-formulations could modify the angiogenic and inflammatory features of melanoma tumors, a screening of 24 proteins was conducted by using RayBio^®^ Mouse Angiogenic protein Antibody Array membranes 1.1 (RayBiotech Inc., Peachtree Corners, GA, United States) coated with 24 primary antibodies specific for mouse proteins of interest. One array membrane per experimental group was incubated overnight at 4°C with 250 µg total protein from each lysate. Subsequently, biotin-conjugated antibodies were added to the membranes as a mixture, followed by incubation at room temperature for 2 h. Next, HRP-conjugated streptavidin was added onto the membranes for two more hours. In between each incubation step, five washing steps were performed with a kit-provided washing buffer. Finally, a mix of two detection buffers was added to the membranes for 1 min, followed by exposure to X-ray films (Kodak, United States) for 2 min and developing the films. The production levels of each protein were quantified using TotalLab Quant Software version 12 for Windows, by measuring the intensity of the spots, in comparison with the positive control spots. Protein levels from each treated experimental group were expressed as a percentage of corresponding protein levels from the untreated control group. The levels of each protein were measured in duplicate (Licarete et al., 2017).

### Immunohistochemical Analysis of Tumor Tissue

The immunohistochemical analysis was performed as previously described (Luput et al., 2018) for the following antigens: CD31, a marker for vascular differentiation, to show the effects of the nano-formulations on the development of new vasculature; VEGF, to stain the invasive tumor cells in the tumor microenvironment (TME); and F4/80, to highlight the mature macrophages associated to the TME. The following dilutions of the primary antibodies were used: anti-CD31 antibody (rabbit IgG anti-mouse CD31, Abcam, ab124432) diluted 1:1,000, anti-F4/80 mouse macrophage receptor (rat IgG anti-mouse F4/80, Bio-Rad, MCA497) diluted 1:250, and anti-VEGF (rabbit IgG polyclonal/anti-mouse VEGF, Sigma-Aldrich, ABS82) diluted 1:400. Then, the slides were blindly investigated by histologists using an Optika trinocular microscope B383-FL with an MDC CCD Camera 2 MP. After microphotography, the images were prepared in Adobe Photoshop CS6 software in order to generate a whole figure. The area of positive immunoreaction was determined by color densitometry plug-in using Image J software. The scoring system used for immunohistochemical reaction was 0.5 (5–20%); 1 (20–40%); 2 (40–60%); 3 (60–80%); and 4 (80–100%).

### Assessment of Oxidative Stress Parameters

The lipid peroxidation marker MDA in all experimental conditions was quantified *via* HPLC, and values normalized to the protein concentration in tumor lysates were expressed as nano-moles MDA/mg protein. To assess the intratumor activity of catalase, we used the Catalase Assay Kit (Sigma Aldrich Chemie, Munich, GER) to determine the peroxidatic function of the enzyme based on the colorimetric reaction with methanol in the presence of H_2_O_2_, with 4-amino-3-hydrazino-5-mercapto-1,2,4-triazole (Purpald) as the chromogen (Wheeler et al., 1990). Absorbance was measured at 540 nm, and values were expressed as units of catalytic activity/mg protein. The total antioxidant capacity (TAC) of melanoma tumors was measured as reported by Erel (2004) and expressed as μmoles nonenzymatic antioxidants (Trolox)/mg protein. In all cases, the samples were determined in duplicate.

### Western Blot Analysis of Apoptosis and Invasion Markers

The method steps were carried out according to a protocol previously described in detail by us (Rauca et al., 2018; Rauca et al., 2019; Rauca et al., 2021). In brief, 5–10 μg proteins from each lysate were separated by SDS-PAGE onto a 10% polyacrylamide gel and immunoblotted against the following primary antibodies, diluted 500-fold: mouse monoclonal IgG anti-mouse Bcl-xL (sc-8392, Santa Cruz Biotechnology, Texas, United States), rabbit polyclonal IgG anti-mouse Bax (2772S, Cell Signaling Technology, Inc., Danvers, United States), and rabbit monoclonal IgG anti-mouse HIF-1*α* (ab179483, Abcam, Cambridge, United Kingdom). As a loading control, *β*-actin was detected using a rabbit polyclonal IgG against mouse *β*-actin (A2103, Merck, Darmstadt, GER) diluted 1000-fold. A goat anti-mouse horseradish peroxidase (HRP)-conjugated IgG (secondary antibody, sc-2005; Santa Cruz Biotechnology, Inc.) diluted 4000-fold was used to detect the bound antibodies. The immunocomplexes were detected using chemiluminescence and X-ray films. Images of X-ray films were obtained by scanning the films, after exposing them to the NC membranes and to developer/fixer solutions. The X-ray films are stored in our laboratory, and the original images of the X-ray films are provided in the supplementary file. The analysis of the films was performed using Image J.JS online version freeware. Data were presented as mean ± standard deviation (SD) of two independent experiments.

### Statistical Analysis

Data originating from separate experiments were presented as mean ± standard deviation (SD). Statistical comparison of the effects of the treatments on tumor growth and oxidative stress parameters, as well as on the levels of expression of different proteins by western blot analysis, was evaluated by one-way ANOVA with Bonferroni correction for multiple comparisons. Statistical analysis of treatment effect on angiogenic/inflammatory protein production was performed by two-way ANOVA followed by Bonferroni correction for multiple comparisons. The intensity scores of immunoreactions in tumor sections were interpreted using the Kruskal–Wallis non-parametric test followed by Dunn’s test for the multiple comparisons. All statistical analyses were performed by using GraphPad Prism 9.2.0.332. A *p* value lower than 0.05 was considered significant.

## Results

This study provides a follow-up of our recently published article (Patras et al., 2021) in which we demonstrated the increased efficacy of functionalized EVs (PEG-EV-DOX) used as systemic delivery tools of DOX in melanoma by specifically targeting tumor cells. By combining the novel IL-13-LCL-SIM formulation with PEG-EV-DOX, we aimed to target both the protumoral M2-like phenotype of TAMs and the developmental capacities of melanoma cells**.** The characterization of PEG-EV-DOX was presented in detail in our previous study (an average size of 117 ± 10.5 nm, average PDI of 0.165 ± 0.07, PEGylation efficiency of 0.1 mol% PEG concentration from total phospholipid mass of EVs, DOX concentration of 455 μg/ml, and encapsulation efficiency of 45.5 ± 15.4%) (Patras et al., 2021). The characterization of the liposomal formulations used in this study is presented in the following.

### Characterization of Nano-Formulations

The physical and chemical properties of liposomal formulations were analyzed regarding particle size distribution, polydispersity index, zeta potential, the concentration of the active drug, and encapsulation efficiency**.** The biological properties of nano-particles were assessed in regard with their stability in simulated biological fluids. As shown in Table 1, all liposomal formulations were characterized as size, polydispersity index, and zeta potential using the Zetasizer Nano ZS (Malvern Instruments, United Kingdom). The particle size of all types of liposomes ranged between 130 and 198 nm, significantly lower than the cutoff limits of tumor vasculature pores (Porfire et al., 2015). The low PDI values (under 0.3) were indicative of homogenous particles (Danaei et al., 2018). Zeta potential values (between –30 and –40 mV) were in the range of stability in colloidal dispersions (Lin et al., 2014). The encapsulation efficiency values of SIM were 50.72% (LCL-SIM) and 65.21% (IL-13-LCL-SIM), values that indicate potential for technological transfer (Table 1). To assess stability in simulated biological fluids, liposomes were tested in HBBS and in FCS to approximate *in vivo* fluid conditions (Sesarman et al., 2021). All LCL formulations were diluted with the mentioned fluids in a ratio of 1:100 (v/v) and incubated at 37°C for 24 and 48 h. The size of the liposomes was measured before (*t* = 0) and after 24 h (*t* = 24) and 48 h (*t* = 48 h) incubation, and results are presented in Table 2. FCS elicited a moderate increase in both IL-13-LCL-SIM and LCL-SIM size (not more than 15% compared to size at *t* = 0, suggesting good colloidal stability (Sesarman et al., 2021). In HBBS, the size of the liposomal formulations presented weak variations at each time point of measurement. In conclusion, our results in Table 1 and Table 2 suggested optimal size, homogenous distribution, and good stability of all liposomal formulations in *in vivo* simulating conditions. The circulation time is a definitory aspect for the biodistribution and therapeutic efficiency of nano-particles. It has already been shown that PEGylation of extracellular vesicles isolated from cancer cell increases *in vivo* circulation time and biodistribution by interfering with systemic particle clearance (Kooijmans et al., 2016). According to this study, PEGylated EVs can remain in plasma in between 60 and 240 min compared to unmodified EVs that are removed after 10 mins, and organ distribution is unaltered between the two types of EVs that mainly accumulate in RES organs, the liver and spleen. In this light, for this study, we used tumor-derived EVs that were decorated with polyethylene glycol-2000 (PEG2000) by the post-insertion method to prevent opsonization or interaction with mononuclear phagocytes in the bloodstream, leading to more efficient tumor and organ distribution (Patras et al., 2021). The IL-13-LCL-SIM liposomal nano-formulation used in this study is also PEGylated, which was shown to lead to extended blood circulation time by creating a steric barrier between liposomes and plasma components, leading to enhanced biodistribution and preferential accumulation to the tumor site *via* the enhanced permeability and retention (EPR) effect (Zhuang et al., 2012). Besides size and charge, the lipid composition is one of the most important factors involved in liposome stability, and cholesterol plays a crucial role in this regard (Nakhaei et al., 2021). Previous studies using more or less similar lipid molar ratios to the IL-13-LCL-SIM formulation that we used (75% DPPC, 15% cholesterol, 5% PEG, and a molar ratio of CHOL-SIM = 2.5:1) already assessed the stability, bioavailability in plasma, and tissue distribution of long-circulating liposomes encapsulating simvastatin or doxorubicin (Tuerdi et al., 2020; Sambamoorthy et al., 2021; Sesarman et al., 2021). Besides the liposomal stability studies in simulated biological fluids (FCS and HBBS) that we already provided in the current manuscript, in another study (Barbălată et al., 2021), we analyzed liposomes containing either SIM or DOX, with the following lipid molar ratios DPPC: MPEG-2000-DSPE: CHO = 95:5:10, in relation to their *in vitro* drug release ability by using the modified dialysis cassette method. Thus, we proved that the acidic pH of the tumor microenvironment determines a two-fold increase of DOX leakage from liposomes compared to the physiological pH and that diffusion process of SIM is delayed, which consequently leads to a slow release rate from the liposomal formulation, unaffecting the cytotoxic properties of the drug. This data come in support of our current sequential treatment scheme with the administration of IL-13-LCL-SIM 24 h in advance. Furthermore, a study by Campos-Martorell et al. (2016) using differently charged liposomes containing 5% PEG to find an optimal delivery system for SIM in ischemic stroke rats reported significant accumulation in the liver, spleen, kidneys, and lungs only in the case of positively charged liposomes compared to negatively charged or neutral liposomes which were distributed homogenously in various tissues. Likewise, neutral and negatively charged liposomes also exhibited longer blood circulation time and higher bioavailability 90 min after administration. This showed that charge is a major factor involved in liposome biodistribution. Their LCL-SIM formulation, although being slightly different than ours regarding component lipids and molar ratio (DLPC 50%:CHOL 45%:CHOL–PEG 5%), presented a neutral-to-negative zeta potential (−1.01 mV compared to −36.3 mV of our IL-13-LCL-SIM nano-formulation) and very similar particle size values (151.85 nm compared to 130 nm of our IL-13-LCL-SIM nano-formulation); thus, we consider this study a good reference for the plasma clearance and biodistribution estimation of our liposomal nano-formulation. In addition, a study performed by our group investigated the pharmacokinetic behavior and tissue distribution of 5% PEGylated liposomes containing DOX and curcumin, with a 10:1 phospholipid:cholesterol molar ratio. This liposomal formulation exceeded the limit of quantification in plasma for up to 4 h after administration. LCL-CURC-DOX, as well as LCL-DOX, reached a peak plasma concentration after approximately 5 min and substantially higher Tmax (time to reach the peak plasma concentration) and t_1/2_ (terminal half-life) compared to free DOX. Moreover, there was no significant increase in the heart and liver distribution of LCL-CURC-DOX compared to free DOX, and no change in relative organ weight was reported as a potential adverse effect (Sesarman et al., 2021).

**TABLE 1:** Characterization of free and SIM-encapsulating LCL formulations.

Liposomal formulation	Size	PDI	Zeta potential (mV)	Therapeutic agent concentration (μg/ml)	Encapsulation efficiency (%)	Phospholipid concentration (mM)
LCL	165.1 ± 2.13	0.022 ± 0.01	−28.8 ± 1.17	—	—	20
IL-13-LCL	198.1 ± 0.40	0.08 ± 0.01	−44.2 ± 0.65	—	—	11
LCL-SIM	148.93 ± 1.09	0.094 ± 0.03	−34.1 ± 0.73	20	50.72	13
IL-13-LCL-SIM	129.9 ± 3.61	0.25 ± 0.13	−36.3 ± 1.09	11	65.21	10

PDI, polydispersity index. Data are presented as mean ± SD of three independent measurements.

**TABLE 2:** Liposome stability.

Biological fluid	Liposomal formulation	Size		
		*t* = 0 h	*t* = 24 h	*t* = 48 h
FCS	IL-13-LCL-SIM	219.6 ± 102.3	242.5 ± 115.2	237.3 ± 127.9
	LCL-SIM	242.7 ± 133.4	228.5 ± 118	251.8 ± 104
HBBS	IL-13-LCL-SIM	181.9 ± 46.87	182.1 ± 41.52	195.2 ± 64.47
	LCL-SIM	162.2 ± 37.57	162.3 ± 35.54	169.8 ± 52.55

FCS, fetal calf serum; HBBS: Hank’s balanced salt solution. Data are presented as mean ± SD of three independent measurements.

### Preferential Uptake of IL-13-Conjugated Long-Circulating Liposomes by M2 Macrophages

**FIGURE 1:**
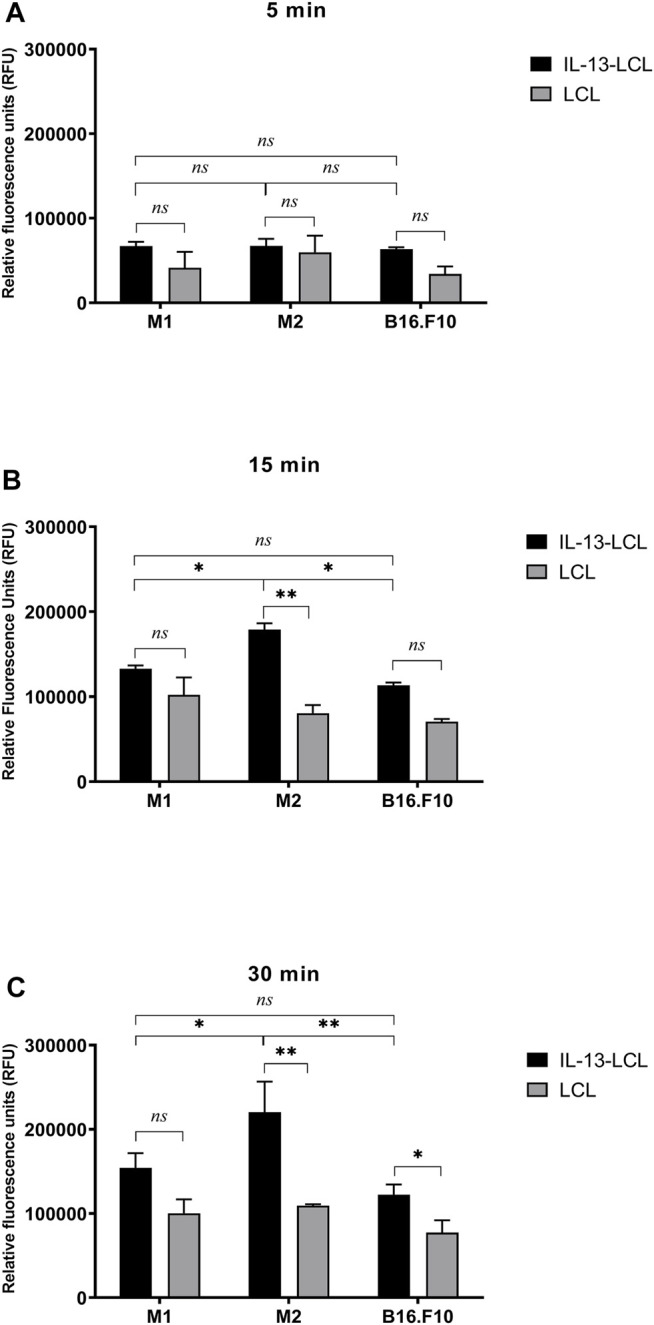
Spectrofluorimetric assessment of IL-13 functionalized LCL uptake by M1/M2 macrophages and B16.F10 cells compared to unconjugated LCL uptake. Uptake measurements were performed after 5 **(A)**, 15 **(B)**, and 30 **(C)** min incubation of all cell types with a concentration of 20 mM (LCL) and 11 mM (IL-13-LCL) phospholipids containing the fluorescent component rhodamine. Results were expressed as mean ± SD of triplicate measurements and shown as Relative Fluorescence Units (RFUs). Untreated M1/M2 polarized macrophages and B16.F10 cells were used to remove background cell autofluorescence. The unpaired t-test was used to compare the uptake of IL-13-LCL and LCL in M1/M2 macrophages and B16.F10 melanoma cells; *ns*—not significant; *p* > 0.05; *, *p* < 0.05; and **, *p* < 0.01.

Quantitative spectrofluorimetric uptake measurements of IL-13-LCL by M1/M2 macrophages and B16.F10 cells were compared to the uptake of unconjugated LCL (Figure 1). After 5 min incubation with the liposomal formulations, there was no significant difference between the uptake of IL-13 and LCL by all cell types (Figure 1A, *p >* 0.05). After 15 min incubation with the liposomal formulations, the superior binding capacity of IL-13-LCL to M2 macrophages compared to M1 macrophages and tumor cells was reported (Figure 1B, *p* < 0.05). Moreover, only in the M2 macrophages, the uptake of IL-13-LCL was significantly higher by 2-fold compared to LCL (Figure 1B, *p* < 0.01). A similar trend was observed after 30 min incubation, IL-13-LCL being preferentially uptaken by M2-polarized macrophages compared to M1-polarized macrophages (Figure 1C, *p* < 0.05) and B16.F10 melanoma cells (Figure 1C, *p* < 0.01).

### Antiproliferative Effects of Sequential Nano-Therapy Consisting of IL-13-Conjugated Long-Circulating Liposomes Containing SIM Targeting Tumor-Associated Macrophages and Doxorubicin Encapsulated Into PEG-Coated Extracellular Vesicles Targeting Melanoma Cells in Co-Cultures

**FIGURE 2:**
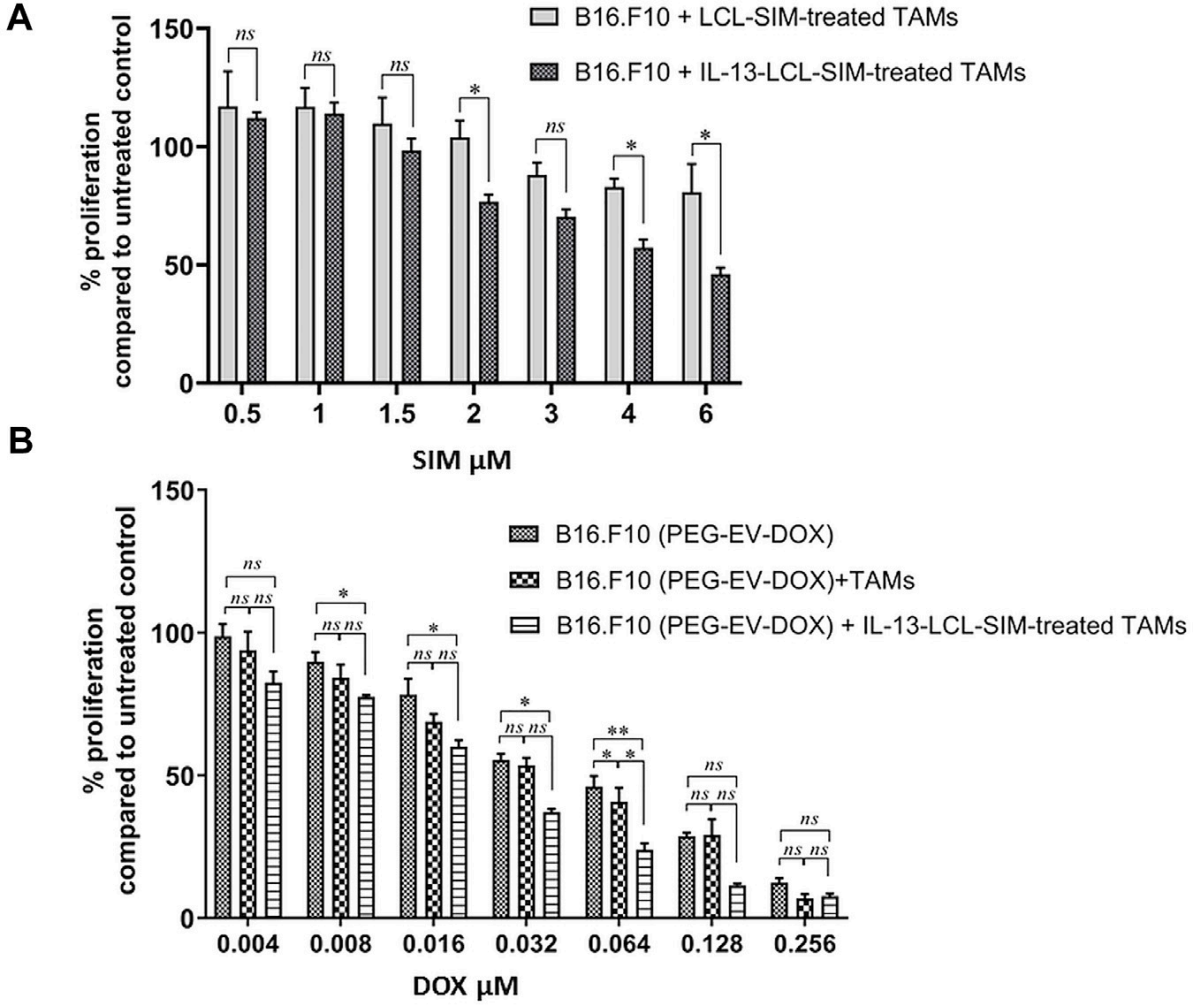
Antiproliferative effects of sequential administration of IL-13-LCL-SIM and PEG-EV-DOX on B16.F10 cell proliferation. **(A)** B16.F10 cell proliferation in co-culture with either LCL-SIM or IL-13-LCL-SIM-treated M2 macrophages (treated with various concentrations 24 h prior to adding both cell types in co-culture). **(B)** Effects of various concentrations of DOX incorporated in PEG-EV-DOX on the proliferation of B16.F10 melanoma cells monocultured, co-cultured with M2 macrophages or with IL-13-LCL-SIM-treated M2 macrophages (treated with 2 μM SIM encapsulated in IL-13-LCL-SIM 24 h prior to adding both cell types in co-culture). The data represent mean ± SD of triplicate measurements. The unpaired *t*-test was used to compare the effects of different treatments to LCL-treated/-untreated B16.F10 controls*; ns—*not significant; *p >* 0.05; *, p < 0.05; and **, p < 0.01.

The proliferative activity of B16.F10 melanoma cells co-cultured with M2 macrophages which were previously treated for 24 h with IL-13-LCL-SIM, LCL-SIM, and LCL devoid of drug (control) was assessed as in Figure 2A. At lower doses of SIM (0.5–1.5 µM) encapsulated in liposomes, there was no significant difference in the effect of IL-13-LCL-SIM- and LCL-SIM-treated macrophages on B16.F10 cell proliferation. At higher doses of SIM (2–6 µM), B16.F10 cell proliferation was overall significantly reduced when co-cultured with IL-13-LCL-SIM-treated M2 macrophages compared to LCL-SIM-treated M2 macrophages (*p* < 0.05), suggesting that the targeted high-affinity treatment induced a better modulation of the capacity of M2 macrophages to support melanoma cell growth (Figure 2A). To test the effects of the sequential therapy consisting of IL-13-LCL-SIM and PEG-EV-DOX on the co-culture between M2 macrophages and B16.F10 cells, we used M2 macrophages treated with Il-13-LCL-SIM (2 µM) for 24 h and M2 untreated macrophages that were co-cultured with B16.F10 cells, and the entire co-culture was consecutively treated with various concentrations of DOX encapsulated in EVs (PEG-EV-DOX) ranging from 0.004 to 0.256 µM for 24 h. Besides untreated cells, B16.F10 cells in the monoculture treated for 24 h with the same concentrations of DOX incorporated in PEG-EV-DOX were used as control (Figure 2B). Our results demonstrated that “sensitizing” M2 macrophages with IL-13-LCL-SIM before co-culturing them with B16.F10 cells boosted the antiproliferative effects of PEG-EV-DOX treatment on the B16.F10-TAMs co-culture. The most effective sequential combination was considered 2 µM SIM encapsulated in IL-13-LCL-SIM + 0.064 µM DOX incorporated in PEG-EV-DOX (Figure 2B, *p* < 0.01). In accordance with our findings, the IC_50_ of various doses of DOX incorporated in PEG-EV-DOX and administered on B16.F10 monoculture, B16.F10 co-cultured with TAMs and B16.F10 co-cultured with IL-13-LCL-SIM-treated TAMs showed a 2-fold reduction (IC_50_ = 0.0674) in the sequential therapy-treated condition compared to the other two experimental conditions (Table 3).

**TABLE 3:** IC_50_ of the treatments on B16.F10 proliferation in co-culture with TAMs.

Condition	IC_50_ (µM)	Confidence interval 95%
B16.F10 (PEG-EV-DOX)	0.1242	0.02059 to 0.08619
B16.F10 (PEG-EV-DOX) + TAMs	0.12846	0.01567 to 0.1279
B16.F10 (PEG-EV-DOX) + TAMs (IL-13-LCL-SIM)	0.0674	0.01274 to 0.03908

IC_50_: the half-maximal inhibitory concentration of PEG-EV-DOX on B16.F10 melanoma cells in monoculture and in co-culture with TAMs untreated or treated with 2 µM SIM encapsulated in IL-13-LCL-SIM 24 h prior to adding both cell types in co-culture.

### Strong Inhibition of Melanoma Tumor Growth by Sequential Administration of IL-13-Conjugated Long-Circulating Liposomes Containing SIM and Doxorubicin Encapsulated Into PEG-Coated Extracellular Vesicles *In Vivo*

**FIGURE 3:**
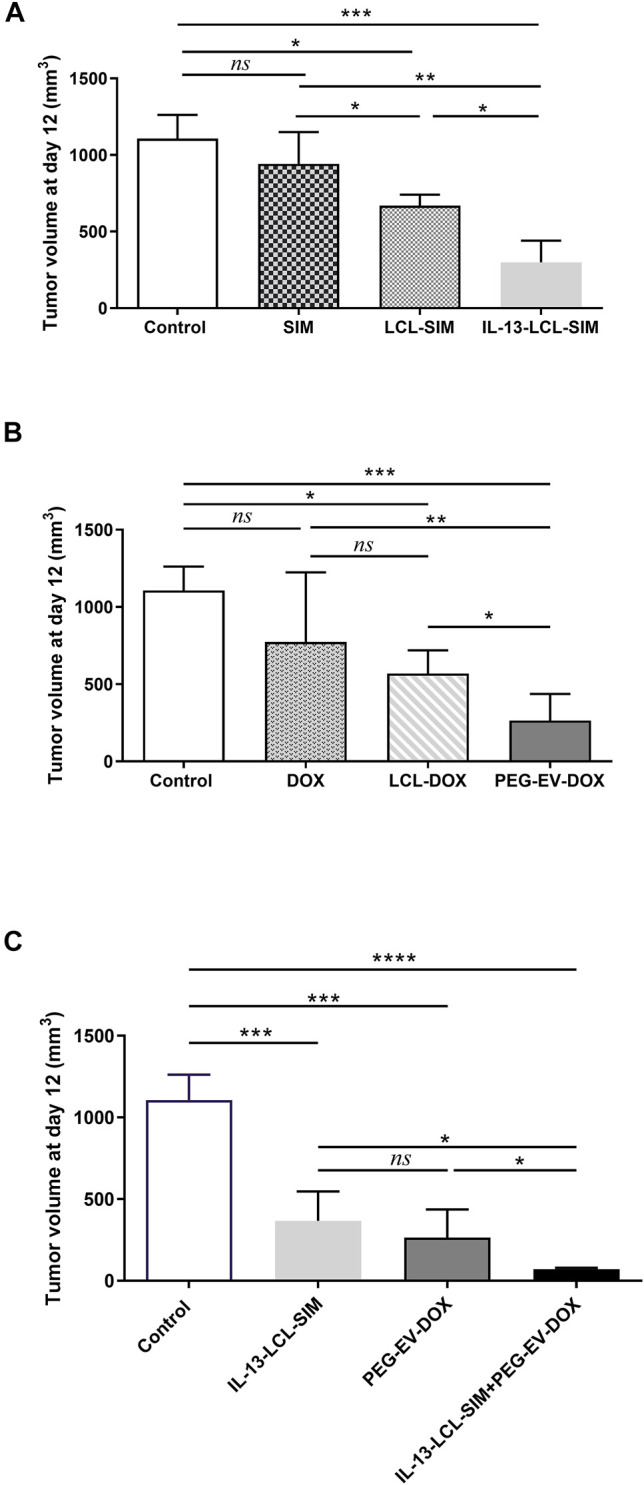
Effects of different treatments on tumor growth. Tumor volumes after different treatments at day 12 (when mice were euthanized) are shown in panels **(A–C)**. Experimental groups (*n* = 5) received two *i.v*. injections containing **(A)** 5 mg/kg SIM in free form and encapsulated in LCL-SIM or IL-13-LCL-SIM on days 7 and 10 after melanoma cell inoculation and **(B)** 2 mg/kg DOX in free form or incorporated in LCL-DOX or PEG-EV-DOX on days 8 and 11 after tumor cell inoculation as previously published (Patras et al., 2021). **(C)** Effects of sequential administration of IL-13-LCL-SIM and PEG-EV-DOX on the growth of *s.c*. B16.F10 murine melanoma. 5 mg/kg IL-13-LCL-SIM was administered as monotherapy on days 7 and 10 after tumor cell inoculation and 2 mg/kg PEG-EV-DOX was administered as monotherapy on days 8 and 11 after tumor cell inoculation. The combination therapy group received both treatments in sequential manner. Results were expressed as mean ± SD of tumor volumes of five mice/experimental group. Control, untreated group; IL-13-LCL-SIM, group treated with 5 mg/kg IL-13-LCL-SIM on days 7 and 10 after tumor cell inoculation; PEG-EV-DOX, group treated with 2 mg/kg PEG-EV-DOX on days 8 and 11 after tumor cell inoculation; and IL-13-LCL-SIM + PEG-EV-DOX, the group received the nano-formulations sequentially, IL-13-LCL-SIM 5 mg/kg on days 7 and 10, respectively, and PEG-EV-DOX 2 mg/kg on days 8 and 11 after tumor cell inoculation. One-way ANOVA test with Bonferroni correction for multiple comparisons was performed to analyze the differences between the effects of the treatments on tumor growth; *ns*, *p* > 0.05; *, *p* < 0.05; **, *p* < 0.01; ***, *p* < 0.001; and ****, *p* < 0.0001.

To confirm the *in vitro* findings regarding the preferential uptake of IL-13-LCL-SIM by M2 macrophages and the efficiency of the sequential therapy consisting of IL-13-LCL-SIM and PEG-EV-DOX in *in vivo* settings, syngeneic C57Bl/6 melanoma-bearing mice (*n* = 5/experimental group) were injected *i.v.* with 5 mg/kg SIM in free form or encapsulated in liposomes conjugated on days 7 and 10 after tumor inoculation and with 2 mg/kg DOX, free or incorporated in PEG-coated EVs on days 8 and 11 after tumor inoculation. On day 7, when therapy was started, the average volume of melanoma tumors was 50 mm^3^. The combination therapy group received both treatments in sequential manner (Supplementary Figure S1). Results are shown in Figure 3 as tumor volumes (mm^3^) at the day of euthanization (day 12) representing mean ± SD of data acquired from five mice/experimental group. Our data revealed that 5 mg/kg SIM encapsulated in IL-13-LCL-SIM elicited the strongest inhibitory effect on B16.F10 melanoma growth compared to the effects of the same concentration of SIM encapsulated in LCL-SIM (by 55% inhibition, *p* < 0.05) and to free SIM (by 68% inhibition, *p* < 0.01) (Figure 3A). The superior effects of 2 mg/kg PEG-EV-DOX compared with LCL-DOX and free DOX on tumor growth have already been published by our group in a recent manuscript (Patras et al., 2021), and the graphic is reproduced in Figure 3B of this manuscript to emphasize the superior antitumor efficiency of both optimized nano-formulations used to develop the novel sequential targeted therapy in this study. Notably, in Figure 3C, the effectiveness of the sequential administration of IL-13-LCL-SIM (administered on days 7 and 10 after tumor cell inoculation) and PEG-EV-DOX (administered on days 8 and 11 after tumor cell inoculation) was demonstrated, by reducing tumor growth almost totally compared to the control untreated group, by 94% (*p* < 0,0001) and having a stronger antitumor effect than each of the single therapies, by 80% reduction compared to the IL-13-LCL-SIM-treated group, and by 73% reduction compared to the PEG-EV-DOX-treated group (*p* < 0.05). These results were consistent with the *in vitro*-reported uptake and proliferation data (Figures 1, 2).

### Sequential Targeted Therapy Based on IL-13-Conjugated Long-Circulating Liposomes Containing SIM and Doxorubicin Encapsulated Into PEG-Coated Extracellular Vesicles Exerted Strong Anti-Angiogenic Effects on B16.F10 Murine Melanoma

**FIGURE 4:**
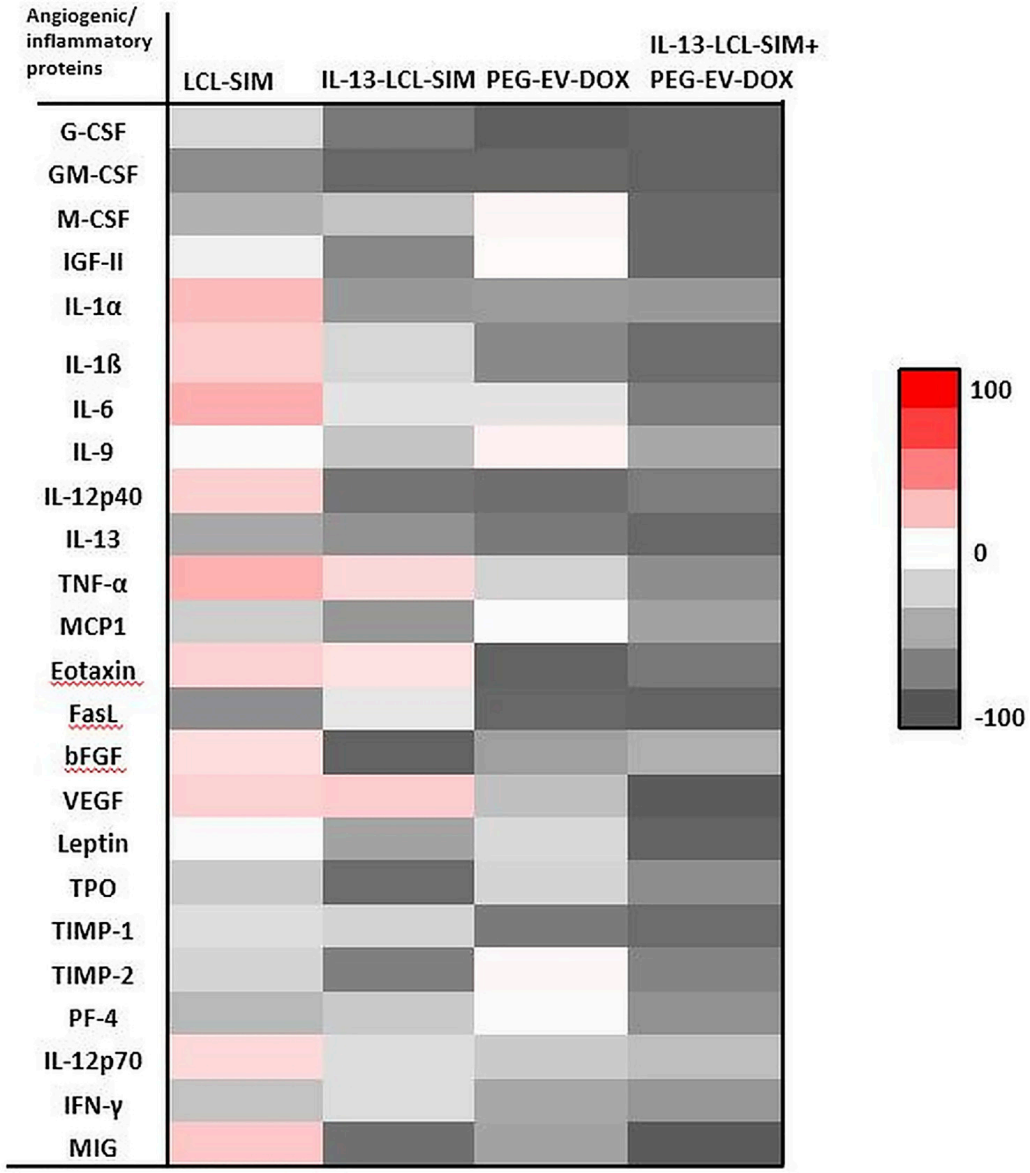
Effects of various treatments on angiogenic protein production in melanoma tumors. Protein levels were compared with the levels of the same proteins in the control group. Data were expressed as average % of reduction (–) of protein levels ranging from 0% (white) to 100% (black) or stimulation (+) of production of proteins ranging from 0% (white) to + 100% (red) compared with the levels of the same proteins in the control group. Control, untreated group; IL-13-LCL-SIM, group treated with 5 mg/kg IL-13-LCL-SIM on days 7 and 10 after tumor cell inoculation; PEG-EV-DOX, group treated with 2 mg/kg PEG-EV-DOX on days 8 and 11 after tumor cell inoculation; IL-13-LCL-SIM + PEG-EV-DOX, the group received the nano-formulations sequentially, IL-13-LCL-SIM 5 mg/kg on days 7 and 10, respectively, and PEG-EV-DOX 2 mg/kg on days 8 and 11 after tumor cell inoculation.

**FIGURE 5:**
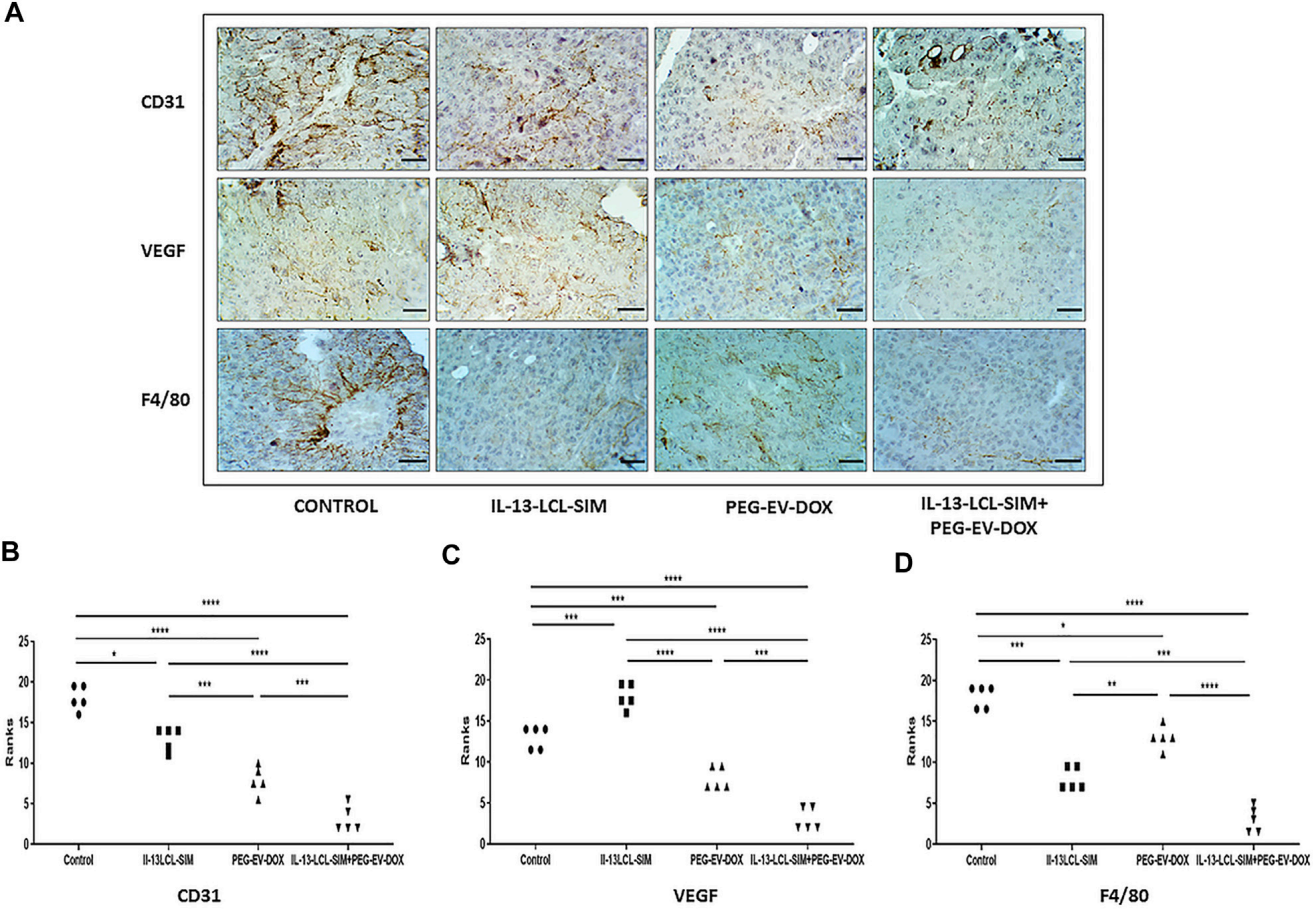
Immunohistochemical analysis of melanoma tissue samples. **(A)** Immunohistochemical imaging of tumor tissues from B16.F10 melanoma-bearing mice after different treatments. Positively stained cells for CD31, a marker for proliferating endothelial cells, for VEGF, the dominant angiogenesis marker, and for F4/80, the pan intratumor murine macrophage marker appear in brown; size bars = 50 μm. **(B–D)** Immunohistochemical scores of angiogenesis markers CD31 **(B)** and VEGF **(C)** and of pan macrophage marker F4/80 (D) in B16.F10 murine melanoma sections treated with IL-13-LCL-SIM and PEG-EV-DOX as monotherapies as well as combined sequential therapy. The immunoreaction intensity scores obtained (minimum 5 random fields/condition) were analyzed by using a rank-based nonparametric Kruskal–Wallis test with Dunn’s test for multiple comparisons. Control, untreated group; IL-13-LCL-SIM, group treated with 5 mg/kg IL-13-LCL-SIM on days 7 and 10 after tumor cell inoculation; PEG-EV-DOX, group treated with 2 mg/kg PEG-EV-DOX on days 8 and 11 after tumor cell inoculation; IL-13-LCL-SIM + PEG-EV-DOX, the group received the nano-formulations sequentially, IL-13-LCL-SIM 5 mg/kg on days 7 and 10, respectively, and PEG-EV-DOX 2 mg/kg on days 8 and 11 after tumor cell inoculation; *, *p* < 0.05; **, *p* < 0.01; ***, *p* < 0.001; and ****, *p* < 0.0001.

As both SIM and DOX induced significant angiogenic effects *in vivo* by nano-formulation-mediated delivery (Licarete et al., 2020; Rauca et al., 2021), we evaluated intratumor angiogenic and inflammatory protein production in whole tumor lysates after administration of 5 mg/kg IL-13-LCL-SIM and 2 mg/kg PEG-EV-DOX as monotherapies and sequential therapy. We also used the 5 mg/kg LCL-SIM-treated experimental group to compare it with the effects of the IL-13-conjugated LCL-SIM. For this, we performed a screening for intratumor levels of 24 angiogenic/inflammatory proteins by protein microarray, and results are shown in Figure 4, Table 4, and Supplementary Figure S2. Additionally, immunohistochemistry analysis regarding the expression of CD31 (endothelial cell proliferation marker), VEGF (dominant melanoma angiogenesis inducer), and F4/80 (pan macrophage marker related to TAMs-induced angiogenesis) (Miettinen et al., 1994; Banciu et al., 2008b; Ge and Ding, 2020) was performed to validate the antiangiogenic activity of IL-13-LCL-SIM and PEG-EV-DOX. The results are shown in Figure 5.

**TABLE 4:** Effects of the nano-formulations on the production of angiogenic and inflammatory proteins in B16/F10 melanoma tumors.

Angiogenic/inflammatory proteins	Percentage of inhibition (−) and stimulation (+) of angiogenic/inflammatory protein production after different treatments compared to the control group			
	LCL-SIM	IL-13-LCL-SIM	PEG-EV-DOX	IL-13-LCL-SIM + PEG-EV-DOX
G-CSF	−23.18 ± 1.34 (*ns*)	−80.03 ± 1.22 (**)	−95.65 ± 2.66 (***)	−91.6249 ± 2.33 (***)
GM-CSF	−67.72 ± 1.58 (*)	−89.93 ± 1.64 (**)	−89.18 ± 1.51 (**)	−93.2073 ± 0.04 (***)
M-CSF	−44.64 ± 0.74 (*ns*)	−34.37 ± 2.10 (*ns*)	3.19 ± 3.22 (*ns*)	−87.9102 ± 0.50 (**)
IGF-II	−8.42 ± 2.01 (*ns*)	−69.97 ± 0.13 (*)	1.77 ± 0.51 (*ns*)	−87.4268 ± 2.29 (**)
IL-1*α*	26.03 ± 10.44 (*ns*)	−60.13 ± 7.43 (*ns*)	−58.78 ± 2.30 (*)	−60.0135 ± 12.37 (ns)
IL-1ß	18.82 ± 2.37 (*ns*)	−23.18 ± 22.96 (*ns*)	−68.98 ± 0.58 (*)	−86.6191 ± 4.32 (**)
IL-6	31.29 ± 1.82 (*ns*)	−15.12 ± 8.35 (*ns*)	−14.79 ± 17.57 (*ns*)	−76.3113 ± 2.31 (*)
IL-9	−0.09 ± 1.66 (*ns*)	−33.19 ± 4.04 (*ns*)	5.30 ± 2.08 (ns)	−50.5792 ± 7.63 (*ns*)
IL-12p40	17.90 ± 5.02 (*ns*)	−81.62 ± 0.05 (**)	−85.14 ± 0.80 (**)	−77.3401 ± 9.41 (*)
IL-13	−51.18 ± 5.67 (*)	−64.10 ± 2.98 (*)	−78.62 ± 9.44 (*)	−89.2541 ± 1.16 (**)
TNF-*α*	30.39 ± 23.66 (*ns*)	15.05 ± 4.90 (*ns*)	−25.25 ± 3.11 (*ns*)	−66.0575 ± 1.63 (*)
MCP1	−28.60 ± 1.90 (*ns*)	−61.98 ± 9.52 (*ns*)	1.12 ± 1.71 (*ns*)	−53.8319 ± 10.26 (ns)
Eotaxin	17.33 ± 2.85 (*ns*)	11.20 ± 9.81 (*ns*)	−92.82 ± 4.80 (***)	−79.553 ± 6.65 (**)
FasL	−66.73 ± 6.61 (**)	−13.71 ± 3.01 (*ns*)	−90.33 ± 1.06 (**)	−92.3357 ± 2.73 (***)
bFGF	12.65 ± 1.47 (*ns*)	−93.25 ± 5.19 (***)	−56.14 ± 9.57 (*ns*)	−46.6898 ± 0.05 (*ns*)
VEGF	17.48 ± 1.63 (*ns*)	19.47 ± 1.33 (*ns*)	−36.14 ± 15.40 (*ns*)	−96.7505 ± 2.54 (***)
Leptin	−1.86 ± 1.76 (*ns*)	−53.03 ± 1.92 (*)	−21.21 ± 1.9 (*ns*)	−92.1563 ± 1.03 (***)
TPO	−30.21 ± 1.13 (*)	−86.83 ± 5.28 (**)	−23.91 ± 9.66 (*ns*)	−66.966 ± 6.34 (*)
TIMP-1	−18.54 ± 5.32 (*ns*)	−24.68 ± 4.68 (*ns*)	−78.18 ± 2.24 (**)	−87.1048 ± 3.30 (**)
TIMP-2	−23.56 ± 11.97 (*ns*)	−75.17 ± 2.20 (**)	2.10 ± 0.32 (*ns*)	−72.1571 ± 4.62 (**)
PF-4	−41.27 ± 4.99 (*)	−30.53 ± 24.34 (*ns*)	−2.34 ± 1.4 (*ns*)	−64.9863 ± 15.97 (*ns*)
IL-12p70	13.89 ± 4.84 (*)	−19.23 ± 3.05 (*ns*)	−29.55 ± 4.25 (*ns*)	−37.7622 ± 15.11 (*ns*)
IFN-*γ*	−35.34 ± 13.52 (*ns*)	−19.90 ± 11.99 (*ns*)	−50.99 ± 6.46 (*ns*)	−61.4396 ± 0.95 (*)
MIG	21.25 ± 4.63 (*ns*)	−84.21 ± 25.63 (*ns*)	−53.23 ± 4.1 (*)	−97.5646 ± 1.89 (***)

The angiogenic/inflammatory protein levels in tumor lysates after different treatments are compared to control– untreated group levels of the same proteins. The results are expressed as % of the average inhibition (−) or stimulation (+) ± SD of two independent measurements. The two-way ANOVA multiple comparison test was used to compare overall effects on the production of pro-/antitumor proteins in tumor lysates from all experimental groups. Control, untreated group; IL-13-LCL-SIM, group treated with 5 mg/kg IL-13-LCL-SIM on days 7 and 10 after tumor cell inoculation; PEG-EV-DOX, group treated with 2 mg/kg PEG-EV-DOX on days 8 and 11 after tumor cell inoculation; IL-13-LCL-SIM + PEG-EV-DOX, the group received the nano-formulations sequentially, IL-13-LCL-SIM 5 mg/kg on days 7 and 10, respectively, and PEG-EV-DOX 2 mg/kg on days 8 and 11 after tumor cell inoculation; (*ns*, *p* > 0.05; *, *p* < 0.05; **, *p* < 0.01; ***, *p* < 0.001).

Our results suggested substantial antiangiogenic effects of IL-13-LCL-SIM and PEG-EV-DOX administered as monotherapies and as sequential therapy (Figure 4; Table 4). The protein production was inhibited to varying degrees by the different treatments, and data were presented as % of protein levels compared to the levels of the same proteins in the control untreated group. LCL-SIM elicited the weakest overall inhibition of angiogenic proteins (from 1 to 66% inhibition of 14 proteins and from 12 to 31% stimulation of 10 proteins). IL-13-LCL-SIM induced a more potent inhibition of angiogenic protein production (13–93% inhibition of 21 proteins and 11–19% stimulation of 3 proteins), indicating a more effective action of the IL-13 conjugated liposomal form. PEG-EV-DOX administered as monotherapy was also very effective in inhibiting the angiogenic protein production (2–95% inhibition of 19 proteins and 1–5% stimulation of 5 proteins). Notably, the sequential administration of IL-13-LCL-SIM and PEG-EV-DOX induced the strongest suppression of angiogenic proteins (37–97% inhibition of all 24 proteins) (Figure 4; Table 4), with VEGF, leptin, FasL, G-CSF, and GM-CSF being almost completely inhibited in comparison to untreated control (>90% inhibition, *p* < 0.001). The violin plot illustrating the distribution of distribution of inhibited/stimulated angiogenic proteins levels (Supplementary Figure S2) confirmed the highest density of strongly inhibited angiogenic proteins (>80% inhibition) in the IL-13-LCL-SIM + PEG-EV-DOX sequential therapy-treated experimental group.

The immunohistochemical analysis of tumor tissue presented confirmatory evidence for the strong antiangiogenic capacity of our novel sequential therapy (Figure 5). The combined therapy induced the strongest inhibition of CD31 expression compared to control (*p* < 0,0001) and to both IL-13-LCL-SIM (*p* < 0,0001) and PEG-EV-DOX (*p* < 0.001) administered as single therapies (Figures 5A,B). IL-13-LCL-SIM monotherapy induced an upregulation in the expression of VEGF (Figures 4, 5A,C; Table 4), presumably secreted by tumor cells as a compensatory mechanism for direct targeting of pro-angiogenic macrophages by IL-13-conjugated liposomes (Li et al., 2016). Notably, the combined sequential therapy was most efficient in reducing VEGF levels compared to both control group (*p* < 0,0001) and PEG-EV-DOX-treated group (*p* < 0.001) (Figures 5 A,C). In accordance with our *in vitro* data showing that IL-13-conjugated liposomes are preferentially taken up by M2 macrophages (Figure 1), IL-13-LCL-SIM monotherapy generated a strong reduction of intratumor macrophage marker F4/80 in the melanoma microenvironment (Figures 5 A,D, *p* < 0.001). PEG-EV-DOX elicited only a weak reduction in F4/80 expression (Figures 5 A,D, *p* < 0.05). Our data suggested that the combination therapy exerted the highest suppression of F4/80 in tumor tissue consistent with the overall highest antiangiogenic efficiency of the sequential combined treatment (Figures 5 A,D).

These findings are a compelling proof of the strong antiangiogenic effects elicited by the combined sequential therapy *via* suppressive actions on major angiogenic proteins, inhibition of vascular endothelial cell proliferation, and simultaneous reduction of pro-angiogenic macrophage density in the melanoma microenvironment.

### Significant Increase in Intratumor Oxidative Stress Induced by IL-13-Conjugated Long-Circulating Liposomes Containing SIM + Doxorubicin Encapsulated Into PEG-Coated Extracellular Vesicles Sequential Therapy

**FIGURE 6:**
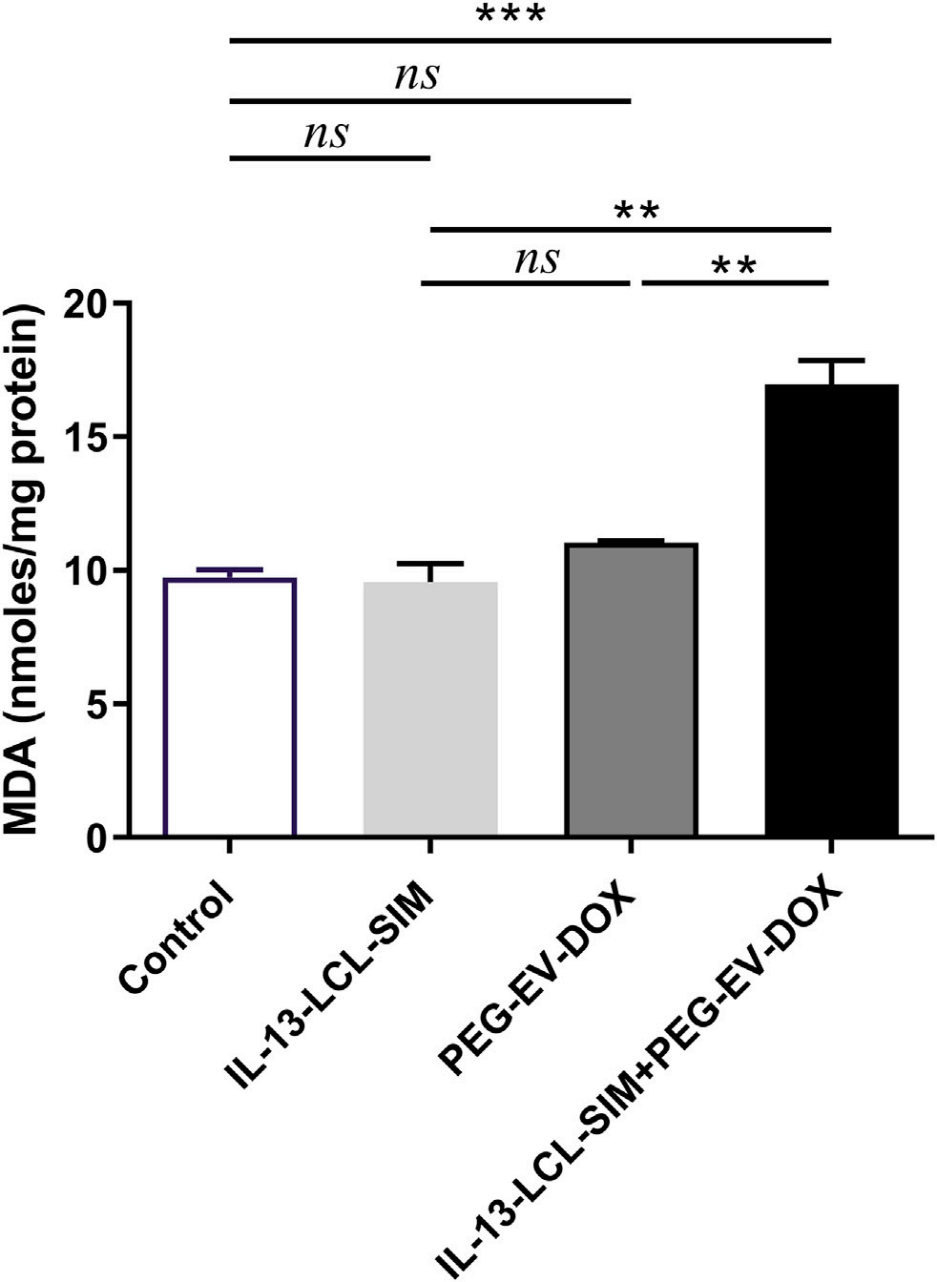
Evaluation of the effects of nano-formulations on oxidative stress biomarker MDA in B16.F10 melanoma. MDA concentration was expressed as nano-moles MDA/mg protein. Measurements were performed in tumor lysates from mice treated with IL-13-LCL-SIM and PEG-EV-DOX as monotherapies as well as combined sequential therapy. Control, untreated group; IL-13-LCL-SIM, group treated with 5 mg/kg IL-13-LCL-SIM on days 7 and 10 after tumor cell inoculation; PEG-EV-DOX, group treated with 2 mg/kg PEG-EV-DOX on days 8 and 11 after tumor cell inoculation; IL-13-LCL-SIM + PEG-EV-DOX, the group received the nano-formulations sequentially, IL-13-LCL-SIM 5 mg/kg on days 7 and 10, respectively, and PEG-EV-DOX 2 mg/kg on days 8 and 11 after tumor cell inoculation; *ns*, *p* > 0.05; **, *p* < 0.01; and ***, *p* < 0.001.

Given the strong antiangiogenic action of the combined therapy, we investigated if these alterations in tumor oxygenation and perfusion could lead to intratumor oxidative stress modulation. Indeed, the sequential combined therapy was able to induce a significant increase in lipid peroxidation product MDA compared to not only control (Figure 6A, *p* < 0.001) but also IL-13-LCL-SIM (Figure 6A, *p* < 0.01) and PEG-EV-DOX (Figure 6A, *p* < 0.01). Although melanoma cells can upregulate their antioxidant scavenging capacity to counteract oxidative stress, resulting in redox metabolic adaptation (Arslanbaeva and Santoro, 2020), our data suggest no increase in enzymatic and non-enzymatic antioxidant mechanisms as a response to the pro-oxidant state induced by IL-13-LCL-SIM and PEG-EV-DOX (Supplementary Figure S3).

Altogether, these results indicate that oxidative stress was increased in the melanoma microenvironment by the combined sequential therapy without triggering compensatory antioxidant protective mechanisms in tumor cells. The overall pro-oxidant state might be attributed to the reported cytotoxic effects of DOX which are induced by generation of reactive oxygen species in targeted cells (Asensio-López et al., 2017; Salvador and Bastos, 2021).

### IL-13-Conjugated Long-Circulating Liposomes Containing SIM + Doxorubicin Encapsulated Into PEG-Coated Extracellular Vesicles Sequential Therapy Drives Apoptotic Switch in the B16 Melanoma Microenvironment

**FIGURE 7:**
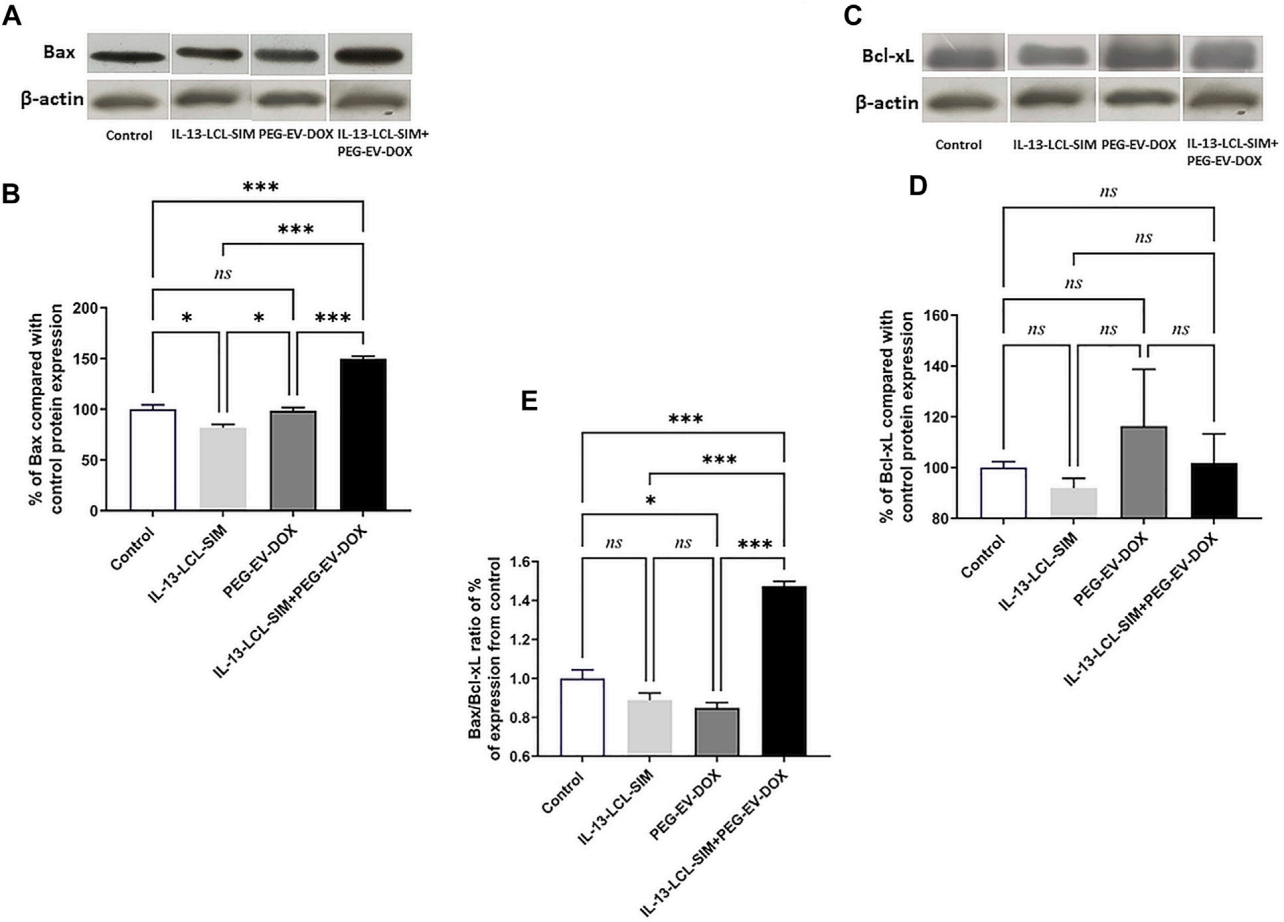
Evaluation of the effect of the treatments on key apoptotic protein levels in TME. The effect of different treatments on intratumor levels of Bax **(A)** and Bcl-xL **(C)** were evaluated by WB. *β*-Actin was used as loading control. The protein levels of Bax **(B)** and BcL-xL **(D)** in tumor lysates were expressed as percentage from the control, untreated group. **(E)** Ratio of % expression levels of Bax/Bcl-xL compared to control. One-way ANOVA test with Bonferroni correction for multiple comparisons was performed to analyze the differences between the effects of the treatments on the apoptotic proteins. Control, untreated group; IL-13-LCL-SIM, group treated with 5 mg/kg IL-13-LCL-SIM on days 7 and 10 after tumor cell inoculation; PEG-EV-DOX, group treated with 2 mg/kg PEG-EV-DOX on days 8 and 11 after tumor cell inoculation; IL-13-LCL-SIM + PEG-EV-DOX, the group received the nano-formulations sequentially, IL-13-LCL-SIM 5 mg/kg on days 7 and 10, respectively, and PEG-EV-DOX 2 mg/kg on days 8 and 11 after tumor cell inoculation; *ns*, *p* > 0.05; *, *p* < 0.05; and ***, *p* < 0.001. The uncropped images of Bax, Bcl-xL, and *β*-actin blots are presented in the supplementary information file.

To investigate if the pro-oxidant state induced by IL-13-LCL-SIM and PEG-EV-DOX could also be related to changes in apoptotic status in the melanoma microenvironment, the relative expression of pro-apoptotic Bax and anti-apoptotic Bcl-xL proteins was assessed *via* WB (Figure 7). Our results have shown that the combined sequential therapy elicited a significant upregulation of pro-apoptotic Bax protein expression (Figure 7A, *p* < 0.001). In contrast, no significant changes in anti-apoptotic Bcl-xL protein expression were detected among experimental conditions (Figure 7B, *p >* 0.05). As previous studies reported that the Bax/Bcl-xL ratio is a valid indicator of melanoma cell sensitivity to therapy by apoptosis induction (Raisova et al., 2001; Kale et al., 2018), we determined this ratio in each experimental condition based on the WB protein expression levels of the two proteins. The results showed about 1.5-fold increase in the Bax/Bcl-xL ratio (Figure 7E, *p* < 0.001) indicating that the combined sequential therapy induced a pro-apoptotic state in the B16 melanoma microenvironment.

To determine the potential of the novel therapeutic approach to target the invasive properties of melanoma cells, we investigated the intratumor production of HIF-1, a major promoter of invasiveness and metastasis in melanoma (Malekan et al., 2021). Our data suggested that there was no difference between HIF-1*α* protein levels in experimental conditions after various treatments compared to the control, untreated group (Supplementary Figure S4).

## Discussion

Systemic delivery of nano-therapeutics in cancer presents the advantage of improved drug bioavailability and reduced systemic toxicological effects due to highly efficient site targeting (Zielińska and Szalata, 2021). The TME plays a compelling role in cancer development, by providing a complex and dynamic stage for bidirectional interactions between tumor and immune cells in favor of progression and metastasis (Baghban et al., 2020). Thus, specifically targeting different key players within the TME at different time points, as well as optimal dosages and frequency of administration, is a crucial factor in achieving maximum drug benefit to the patient (Moradi Kashkooli and Soltani, 2021). TAMs are the dominant immune cell type in the TME, representing up to 30–50% of tumor tissue and playing major roles in tumor angiogenesis growth, invasion, and immunosuppression (Banciu et al., 2008c; Patras et al., 2016; Luput et al., 2017; Rauca et al., 2018; Licarete et al., 2019; Ge and Ding, 2020; Licarete et al., 2020). In tight connection with these findings, our previous studies have shown that the protumor functions of TAMs, as well as the invasive properties of cancer cells, can be impaired by combined therapies delivered *via* optimally designed nano-particles (Banciu et al., 2008b; Alupei et al., 2015; Luput et al., 2018; Sesarman et al., 2018; Rauca et al., 2021). In this context, the current study explored the efficacy of a novel targeted therapy consisting of the sequential administration of IL-13-functionalized long-circulating liposomes encapsulating SIM and EVs stabilized with PEG and loaded with doxorubicin (DOX) (Patras et al., 2021) on B16 melanoma.

Our data showed that the IL-4 polarized M2 macrophages, which express a phenotype similar to the “M2-like” phenotype of TAMs (Ge and Ding, 2020), were the most efficient in taking up the IL-13-LCL *in vitro*, compared to M1 (antitumoral) macrophages and to B16.F10 melanoma cells (Figure 1). Regarding the dynamics of IL-13-LCL uptake, our spectrofluorimetric assessment indicated that already after 15 min of incubation with the liposomal formulations, the M2 macrophage uptake was significantly higher than in the case of M1 and B16.F10 (Figure 1B **)**, suggesting a higher affinity of IL-13 ligand to M2 macrophage surface receptors. We previously demonstrated that TAMs can increase drug resistance when co-cultured with melanoma cells, as higher doses of therapeutic agents were needed to inhibit B16.F10 proliferation in co-cultures (Rauca et al., 2018). Accordingly, IL-13-LCL-SIM treatment on M2 macrophages prior to being co-cultured with melanoma cells slightly decreased their pro-proliferative functions, more efficiently than LCL-SIM (Figure 2A). Moreover, the co-culture between melanoma cells and IL-13-LCL-SIM-treated M2 macrophages rendered B16.F10 melanoma cells more susceptible to the antiproliferative effects of PEG-EV-DOX (Figure 2B), which was also confirmed by the 2-fold decrease in the IC_50_ of PEG-EV-DOX-treated B16.F10 proliferation in the presence of IL-13-LCL-SIM treated TAMs (Table 3). Based on these results and on previous studies in which we demonstrated that liposomal SIM can sensitize cancer cells to cytotoxic agents (Luput et al., 2020) or that intrinsic DOX resistance in melanoma can be overcome by simultaneous administration of inhibitors of TAM pro-tumor functions (Licarete et al., 2020), we administered the novel combination therapy *in vivo*, sequentially as detailed in Supplementary Figure S1. Our data clearly showed that among the monotherapies, IL-13-conjugated LCL-SIM and PEG-EV-DOX elicited the strongest reduction in tumor volume (Figures 3A,B). Notably, IL-13-LCL-SIM and PEG-EV-DOX administered sequentially inhibited almost totally the growth of B16.F10 melanoma tumors, at day 12, when mice were euthanized (Figure 3C **)**. This was in line with our *in vitro* data, and it showed that if we first targeted TAMs *via* IL-13-LCL-SIM, the tumors were much more sensitive to the PEG-EV-DOX treatment. We further showed that the drastic reduction in tumor growth induced by the combined sequential therapy was accompanied by a highly potent suppression of TME angiogenic capacity (Figures 4, 5; Table 4; Supplementary Figure S2). This was based on strong inhibitory actions (>90%) on VEGF (major angiogenesis coordinator), FasL (inducer of anticancer T-cell apoptosis at the vascular border), and leptin (stimulator of angiogenesis and overall accelerator of melanoma growth) (Figures 4, 5A,C; Table 4) (Apte et al., 2019; Su et al., 2020). In addition, the expression level of the CD31 marker of endothelial cell proliferation was also strongly inhibited (Figures 5A,B) and the macrophage density in melanoma tissue was significantly reduced in both IL-13-LCL-SIM and combination therapy groups, indicating an IL-13-LCL-SIM-mediated suppression of angiogenesis-promoting function of TAMs (Figures 5A,D). In our previous study (Rauca et al., 2021), the same concentration of SIM (5 mg/kg) encapsulated in LCL induced a more potent reduction of the angiogenic protein levels in B16.F10 melanoma compared to the current study (Figure 4; Table 4). The differences can be explained by different experimental timelines in relation to the size of the tumors. In the previous experiment, tumors were collected for further analyses on day 15, in the current study at day 12, which could have led to this apparent discrepancy regarding the anti-angiogenic effects of LCL-SIM.

As HIF-1 is known to play a direct role in VEGF-mediated angiogenesis (Malekan et al., 2021) and several of our studies have reported strong inhibitory action of SIM on this important transcriptional regulator in tumor cells (Licarete et al., 2017; Rauca et al., 2018; Rauca et al., 2021), we investigated by WB whether our novel sequential therapy induced a similar effect. However, none of the tested treatments induced significant modifications in the expression levels of HIF-1*α* compared to the control untreated group (Supplementary Figure S4). This finding might come in support of our data regarding the high affinity of IL-13-LCL-SIM for TAMs instead of tumor cells (Figures 1B,C, 2A,2B, 3A,D), which would explain why the protein expression levels of HIF-1*α* remained unchanged among all experimental conditions. Taking these aspects into consideration and having previously reported the preferential uptake of PEG-EV by B16.F10 melanoma cells compared to LCL (Patras et al., 2021), we investigated whether in the case of our therapy the cytotoxic effects on tumor cells could be attributed to PEG-EV-DOX after abrogation of TAMs pro-tumor functions by IL-13-LCL-SIM.

DOX cytotoxic effects are induced by inner mitochondrial membrane localization and ROS production which enhances intracellular toxicity (Montalvo et al., 2020). In melanoma, DOX is reported to induce cell cycle arrest, oxidative stress, and apoptosis if administered in combination with other factors that overcome intrinsic DOX resistance (Licarete et al., 2020; Salvador and Bastos, 2021). Therefore, we next investigated the effects of the targeted sequential therapy consisting of IL-13-LCL-SIM and PEG-EV-DOX on oxidative stress parameters and apoptotic status in the TME. Our results showed both an increase in lipid peroxidation marker MDA levels (Figure 6 **)** and a 1.5-fold increase in Bax/Bcl-xL ratio (Figure 7E) indicating an apoptosis switch triggered in melanoma tissue by the sequential treatment.

## Conclusion

The novel sequential targeted therapy proposed by this study succeeded to both passively and actively target the B16.F10 melanoma microenvironment, leading to almost complete suppression of tumor growth. Thus, IL-13-functionalized LCL-SIM specifically targeted the pro-angiogenic functions of TAMs, downregulated the expression levels of major proangiogenic proteins (VEGF, leptin, FasL) and further sensitized the tumor microenvironment to the cytotoxic effects of PEG-EV-DOX manifested *via* Bax-induced pro-apoptotic status and oxidative stress. The functionalized nano-particles designed for this study could further be exploited in different cancer settings regarding their active tumor targeting potential, circulation time, and clearance mechanisms.

## Data Availability

The original contributions presented in the study are included in the article/Supplementary Material, further inquiries can be directed to the corresponding author.

## Glossary

ANOVA: Analysis of variance
Bax: Bcl2-associated protein X
Bcl-xL: B-cell lymphoma-extra large
bFGF: Basic fibroblast growth factor
BMDMs: Bone marrow-derived macrophages
BrdU: 5-Bromo-2′-deoxyuridine
CD31: Cluster of differentiation 31
CHL: Cholesterol
DMEM: Dulbecco’s modified Eagle’s medium
DMXAA: 5,6-Dimethylxanthenone-4-acetic acid
DOX: Doxorubicin; DPPC, 1,2-dipalmitoyl-sn-glycero-3-phosphocholine
DSPE-PEG-2000-MI: (N-(carbonyl-methoxypolyethylene-glycol-2000)-1,2-distearoyl-sn-glycero-3-phosphoethanolamine, sodium salt)-maleimide
EPR: Enhanced permeability and retention effect
EVs: Extracellular vesicles
F4/80: EGF-like module-containing mucin-like hormone receptor-like 1
FasL: Fas ligand
FCS: Fetal calf serum
G-CSF: Granulocyte colony-stimulating factor
GM-CSF: Granulocyte-macrophage colony-stimulating factor
HBBS: Hank’s balanced salt solution
HIF-1(*α*): Hypoxia-inducible factor 1(*α*)
HPLC: High-performance liquid chromatography
HRP: Horse radish peroxidase
*i.v*: Intravenous
IFN-*γ*: Interferon gamma
IGF-II: Insulin-like growth factor 2
IL-1: Interleukin 1
IL-12p40: Interleukin 12 p40
IL-12p70: Interleukin 12 p70
IL-13: Interleukin 13
IL-13R*α*1: Interleukin-13 receptor subunit alpha-1
IL-13R*α*2: Interleukin-13 receptor subunit alpha-2
IL-1ß: Interleukin 1 ß
IL-1*α*: Interleukin 1*α*
IL-4: Interleukin 4
IL-6: Interleukin 6
IL-8: Interleukin 8
IL-9: Interleukin 9
LCL: Long-circulating liposomes
LPS: Lipopolysaccharide
M1: Macrophages type 1
M2: Macrophages type 2
MCP1: Monocyte chemoattractant protein-1
M-CSF: Macrophage colony-stimulating factor
MDA: Malondialdehyde
MIG: Monokine induced by gamma
MMPs: Matrix metalloproteinases
NC: Nitrocellulose
NO: Nitric oxide
PBS: Phosphate-buffered saline
PDI: Polydispersity index
PEG: Polyethylene glycol
PEG-2000-DSPE: (N-(carbonyl-methoxypolyethylene-glycol-2000)-1,2-distearoyl-sn-glycero-3-phosphoethanolamine, sodium salt)
PEG-EVs-DOX: PEG-coated extracellular vesicles
PF-4: Platelet factor 4
PLP: Prednisolone disodium phosphate
RFU: Relative fluorescence units
Rhodamine B: L-*α*-phosphatidylethanolamine-N-(lissamine rhodamine B sulfonyl) (ammonium salt)
ROS: Reactive oxygen species
*s.c*: Subcutaneous
SD: Standard deviation
SDS-PAGE: Sodium dodecyl-sulfate polyacrylamide gel electrophoresis
SIM: Simvastatin
TAC: Total antioxidant capacity
TAMs: Tumor-associated macrophages
Th2: T-helper type 2 cells
TIMP-1: Tissue inhibitor 1 of metallopeptidase
TIMP-2: Tissue inhibitor 2 of metallopeptidase
TME: Tumor microenvironment
TNF-*α*: Tumor necrosis factor *α*
TPO: Thrombopoietin
VEGF: Vascular endothelial growth factor
WB: Western blot.
