# Supplementary material for: Active Tumor-Targeting Nano-formulations Containing Simvastatin and Doxorubicin Inhibit Melanoma Growth and Angiogenesis
Source: Front Pharmacol. 2022 Apr 5;13:870347. doi: 10.3389/fphar.2022.870347 (PMC9016200; doi:10.3389/fphar.2022.870347)
Supplement: Supplementary file 1 [file Presentation1.PPTX]

## Slide 1
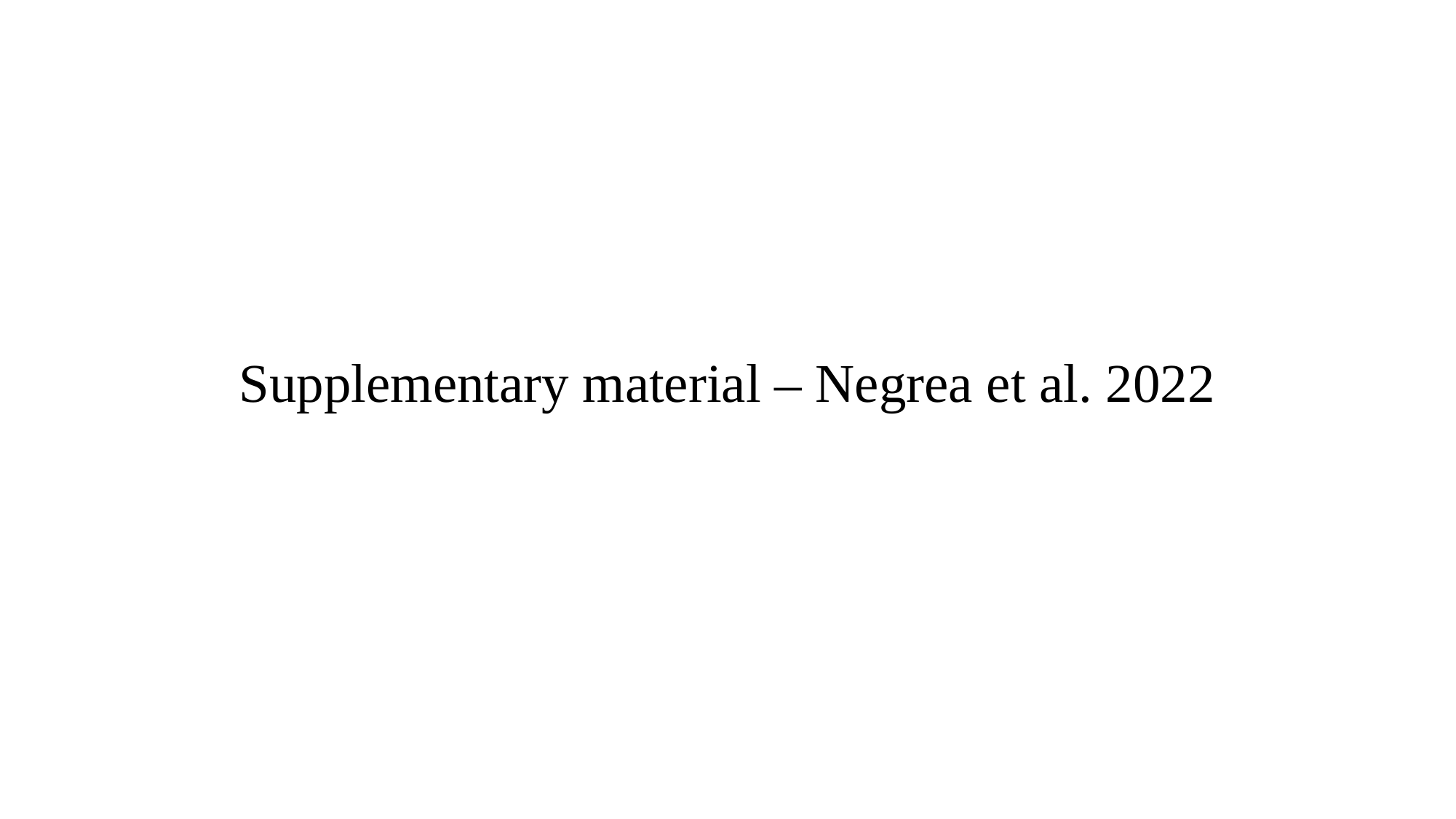

# Supplementary material – Negrea et al. 2022

## Slide 2
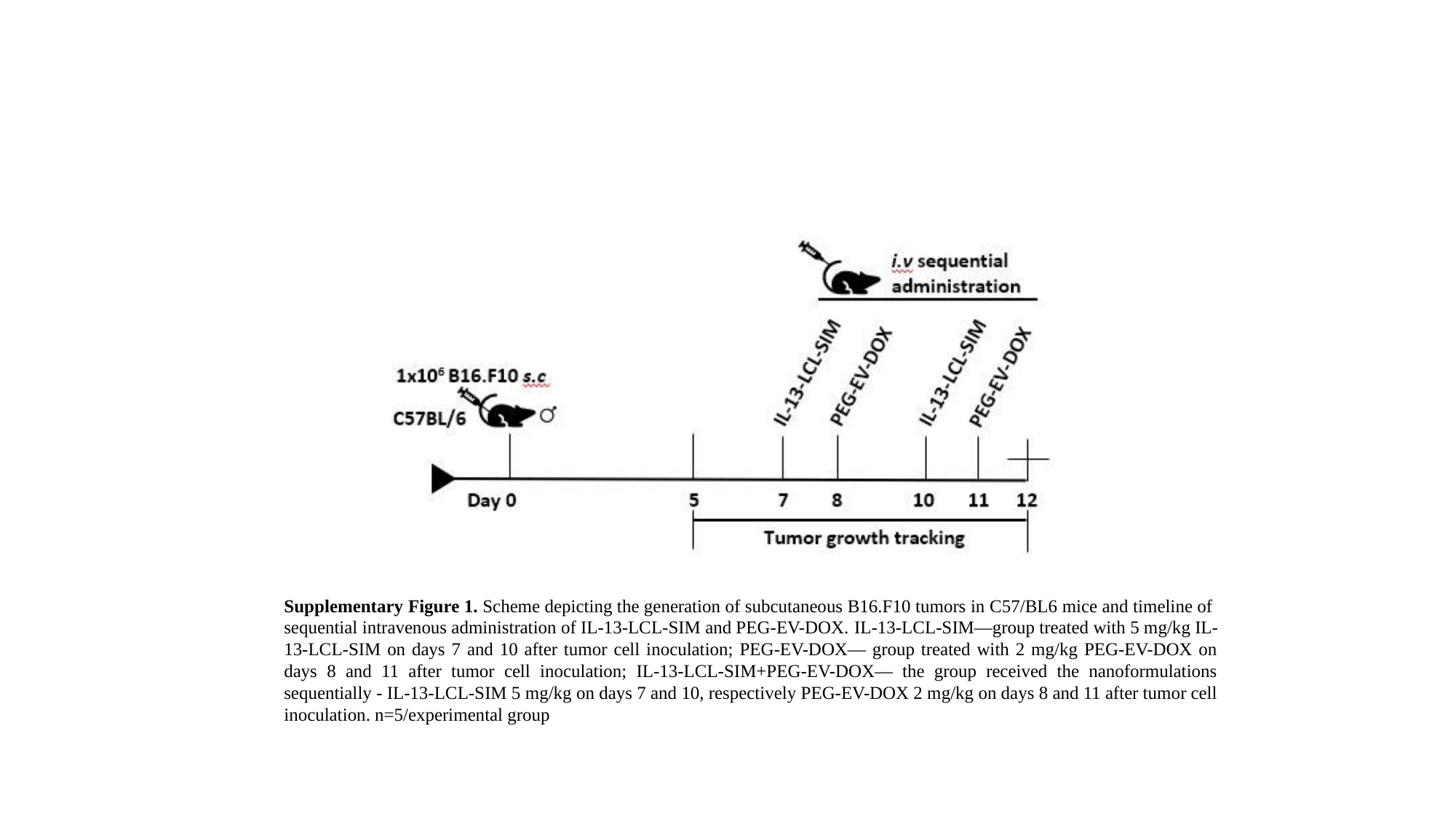

Supplementary Figure 1. Scheme depicting the generation of subcutaneous B16.F10 tumors in C57/BL6 mice and timeline of sequential intravenous administration of IL-13-LCL-SIM and PEG-EV-DOX. IL-13-LCL-SIM—group treated with 5 mg/kg IL-13-LCL-SIM on days 7 and 10 after tumor cell inoculation; PEG-EV-DOX— group treated with 2 mg/kg PEG-EV-DOX on days 8 and 11 after tumor cell inoculation; IL-13-LCL-SIM+PEG-EV-DOX— the group received the nanoformulations sequentially - IL-13-LCL-SIM 5 mg/kg on days 7 and 10, respectively PEG-EV-DOX 2 mg/kg on days 8 and 11 after tumor cell inoculation. n=5/experimental group

## Slide 3
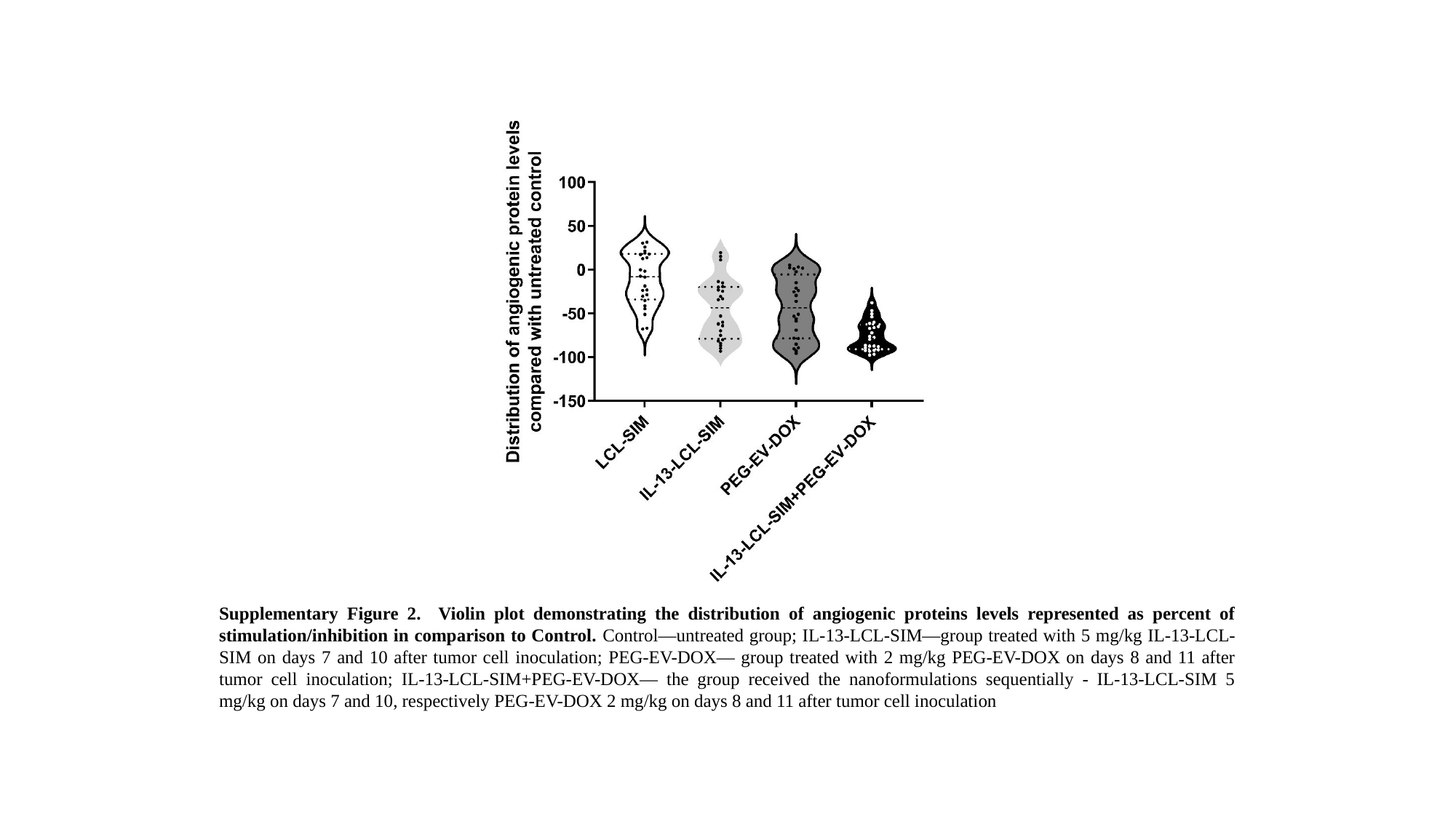

Supplementary Figure 2. Violin plot demonstrating the distribution of angiogenic proteins levels represented as percent of stimulation/inhibition in comparison to Control. Control—untreated group; IL-13-LCL-SIM—group treated with 5 mg/kg IL-13-LCL-SIM on days 7 and 10 after tumor cell inoculation; PEG-EV-DOX— group treated with 2 mg/kg PEG-EV-DOX on days 8 and 11 after tumor cell inoculation; IL-13-LCL-SIM+PEG-EV-DOX— the group received the nanoformulations sequentially - IL-13-LCL-SIM 5 mg/kg on days 7 and 10, respectively PEG-EV-DOX 2 mg/kg on days 8 and 11 after tumor cell inoculation

## Slide 4
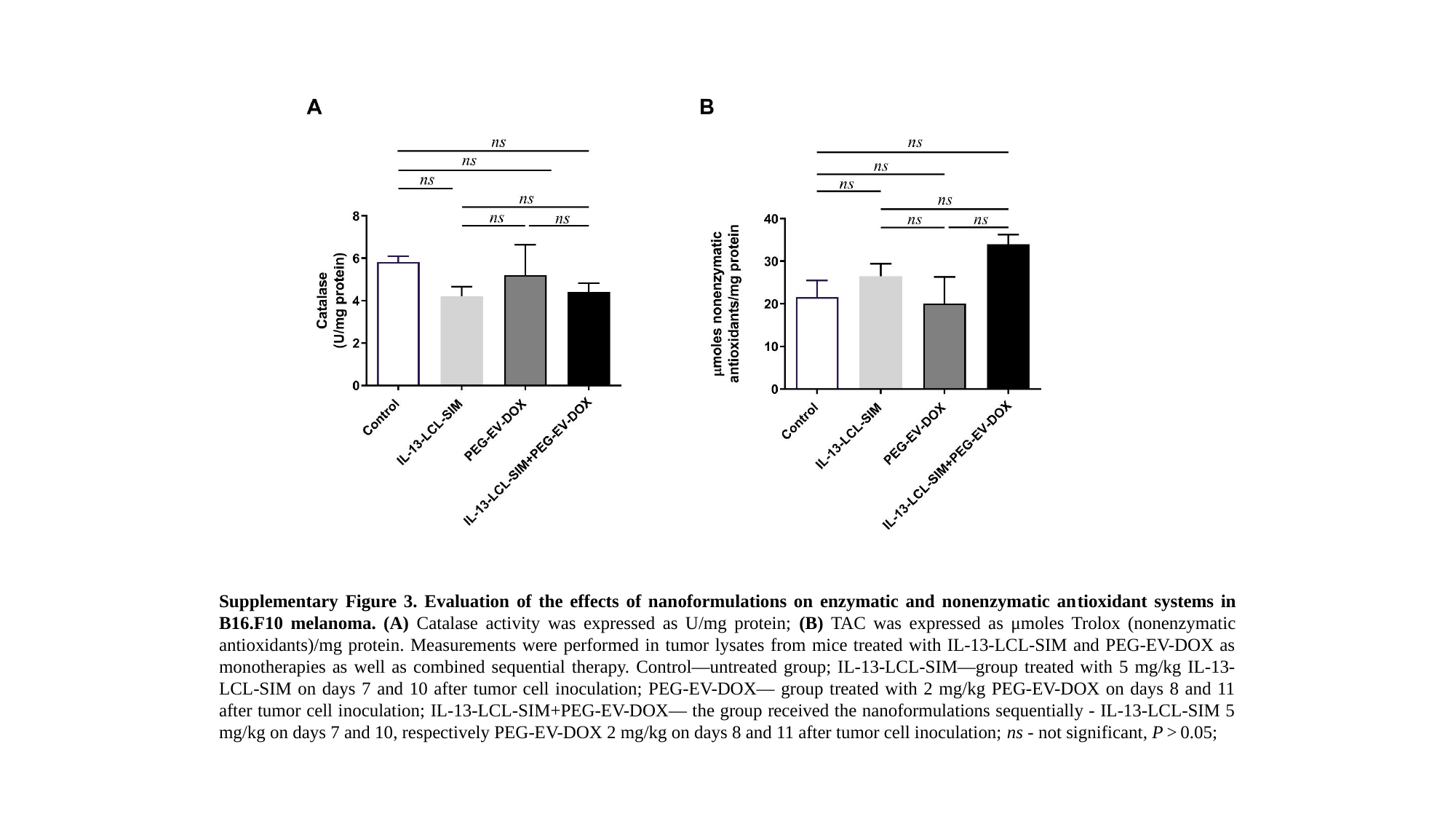

Supplementary Figure 3. Evaluation of the effects of nanoformulations on enzymatic and nonenzymatic antioxidant systems in B16.F10 melanoma. (A) Catalase activity was expressed as U/mg protein; (B) TAC was expressed as μmoles Trolox (nonenzymatic antioxidants)/mg protein. Measurements were performed in tumor lysates from mice treated with IL-13-LCL-SIM and PEG-EV-DOX as monotherapies as well as combined sequential therapy. Control—untreated group; IL-13-LCL-SIM—group treated with 5 mg/kg IL-13-LCL-SIM on days 7 and 10 after tumor cell inoculation; PEG-EV-DOX— group treated with 2 mg/kg PEG-EV-DOX on days 8 and 11 after tumor cell inoculation; IL-13-LCL-SIM+PEG-EV-DOX— the group received the nanoformulations sequentially - IL-13-LCL-SIM 5 mg/kg on days 7 and 10, respectively PEG-EV-DOX 2 mg/kg on days 8 and 11 after tumor cell inoculation; ns - not significant, P > 0.05;

## Slide 5
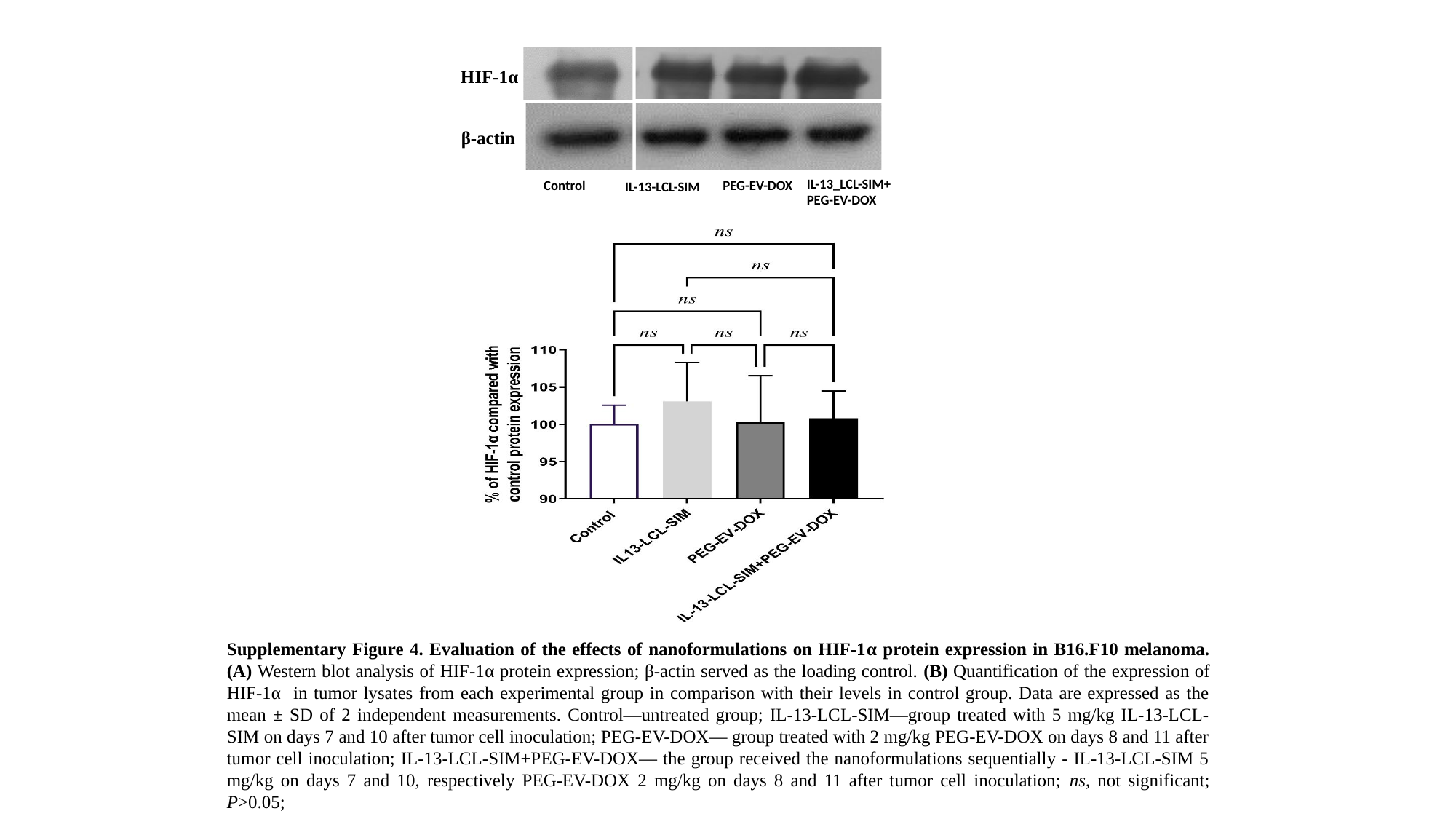

HIF-1α
β-actin
IL-13_LCL-SIM+
PEG-EV-DOX
 Control
PEG-EV-DOX
IL-13-LCL-SIM
Supplementary Figure 4. Evaluation of the effects of nanoformulations on HIF-1α protein expression in B16.F10 melanoma. (A) Western blot analysis of HIF-1α protein expression; β-actin served as the loading control. (B) Quantification of the expression of HIF-1α in tumor lysates from each experimental group in comparison with their levels in control group. Data are expressed as the mean ± SD of 2 independent measurements. Control—untreated group; IL-13-LCL-SIM—group treated with 5 mg/kg IL-13-LCL-SIM on days 7 and 10 after tumor cell inoculation; PEG-EV-DOX— group treated with 2 mg/kg PEG-EV-DOX on days 8 and 11 after tumor cell inoculation; IL-13-LCL-SIM+PEG-EV-DOX— the group received the nanoformulations sequentially - IL-13-LCL-SIM 5 mg/kg on days 7 and 10, respectively PEG-EV-DOX 2 mg/kg on days 8 and 11 after tumor cell inoculation; ns, not significant; P>0.05;

## Slide 6
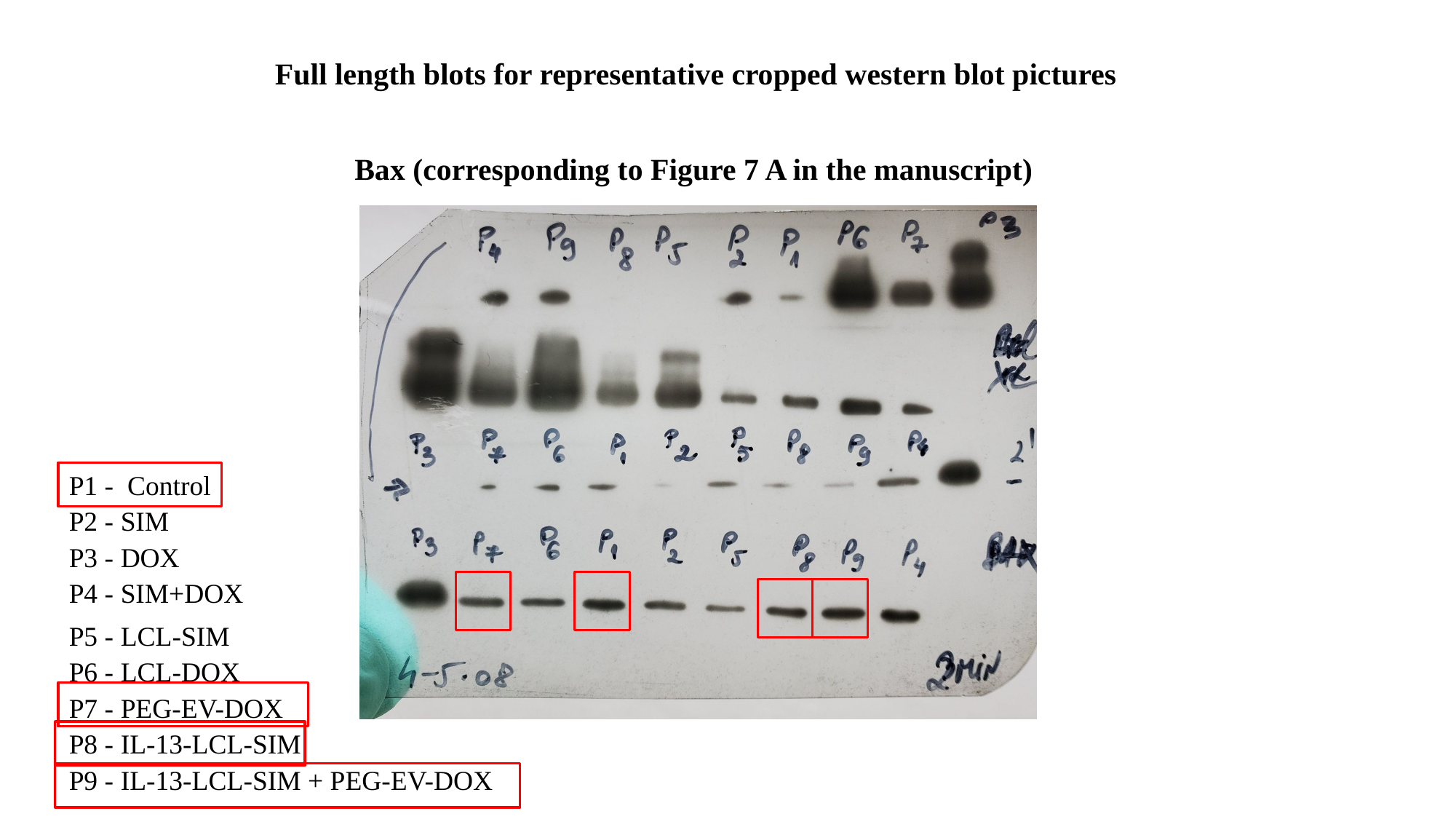

Full length blots for representative cropped western blot pictures
Bax (corresponding to Figure 7 A in the manuscript)
P1 - Control
P2 - SIM
P3 - DOX
P4 - SIM+DOX
P5 - LCL-SIM
P6 - LCL-DOX
P7 - PEG-EV-DOX
P8 - IL-13-LCL-SIM
P9 - IL-13-LCL-SIM + PEG-EV-DOX

## Slide 7
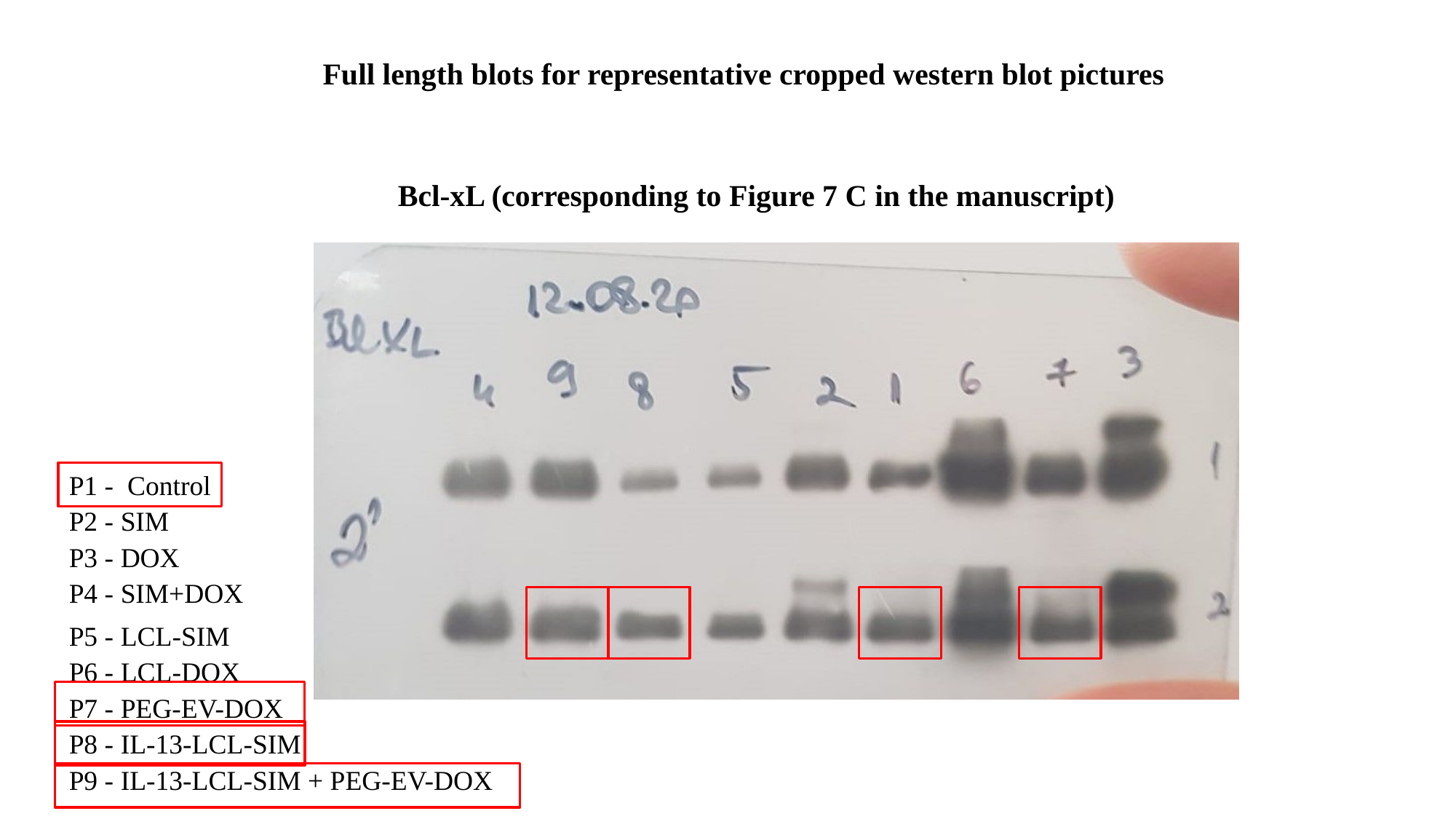

Full length blots for representative cropped western blot pictures
Bcl-xL (corresponding to Figure 7 C in the manuscript)
P1 - Control
P2 - SIM
P3 - DOX
P4 - SIM+DOX
P5 - LCL-SIM
P6 - LCL-DOX
P7 - PEG-EV-DOX
P8 - IL-13-LCL-SIM
P9 - IL-13-LCL-SIM + PEG-EV-DOX

## Slide 8
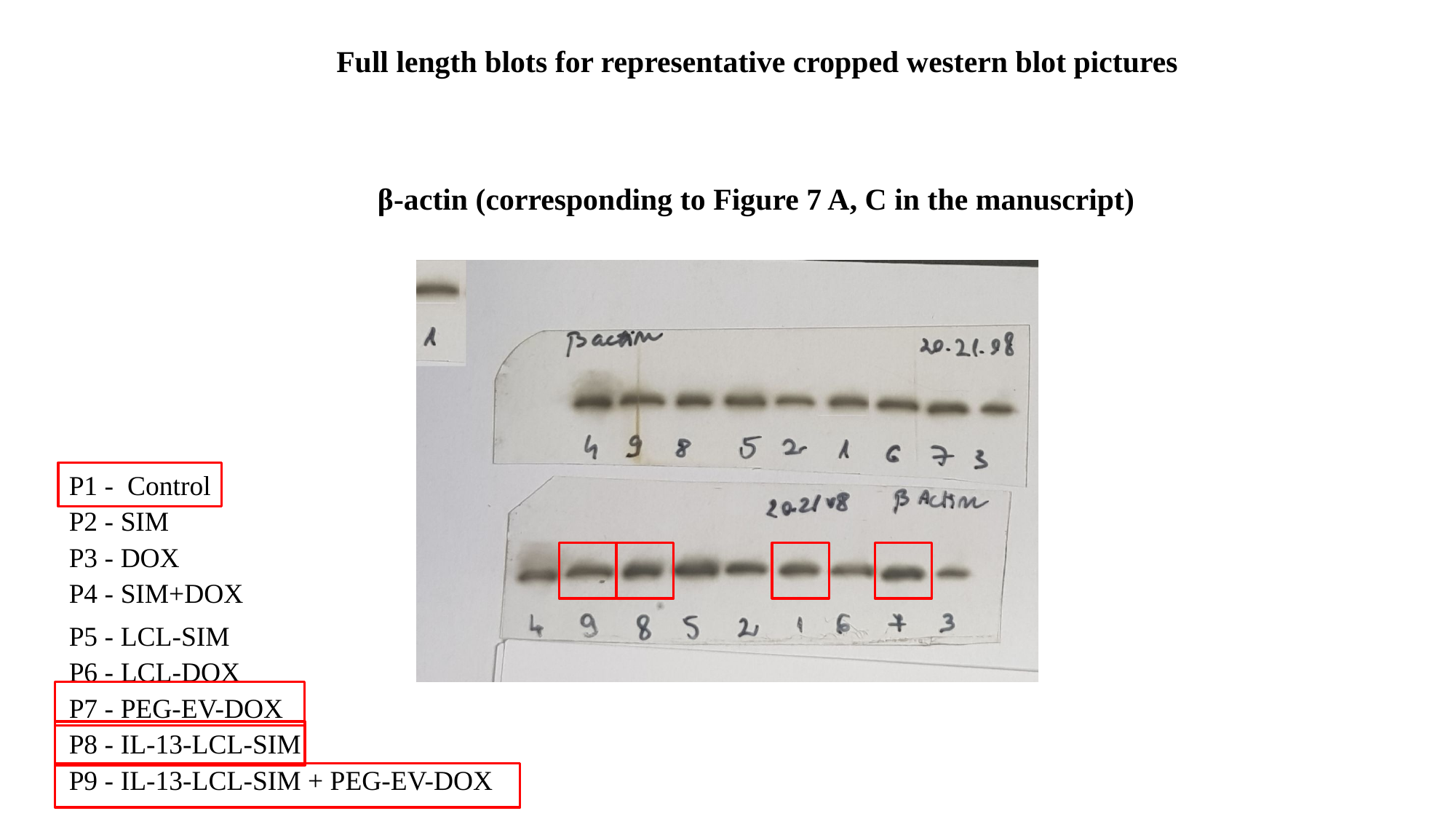

Full length blots for representative cropped western blot pictures
β-actin (corresponding to Figure 7 A, C in the manuscript)
P1 - Control
P2 - SIM
P3 - DOX
P4 - SIM+DOX
P5 - LCL-SIM
P6 - LCL-DOX
P7 - PEG-EV-DOX
P8 - IL-13-LCL-SIM
P9 - IL-13-LCL-SIM + PEG-EV-DOX

## Slide 9
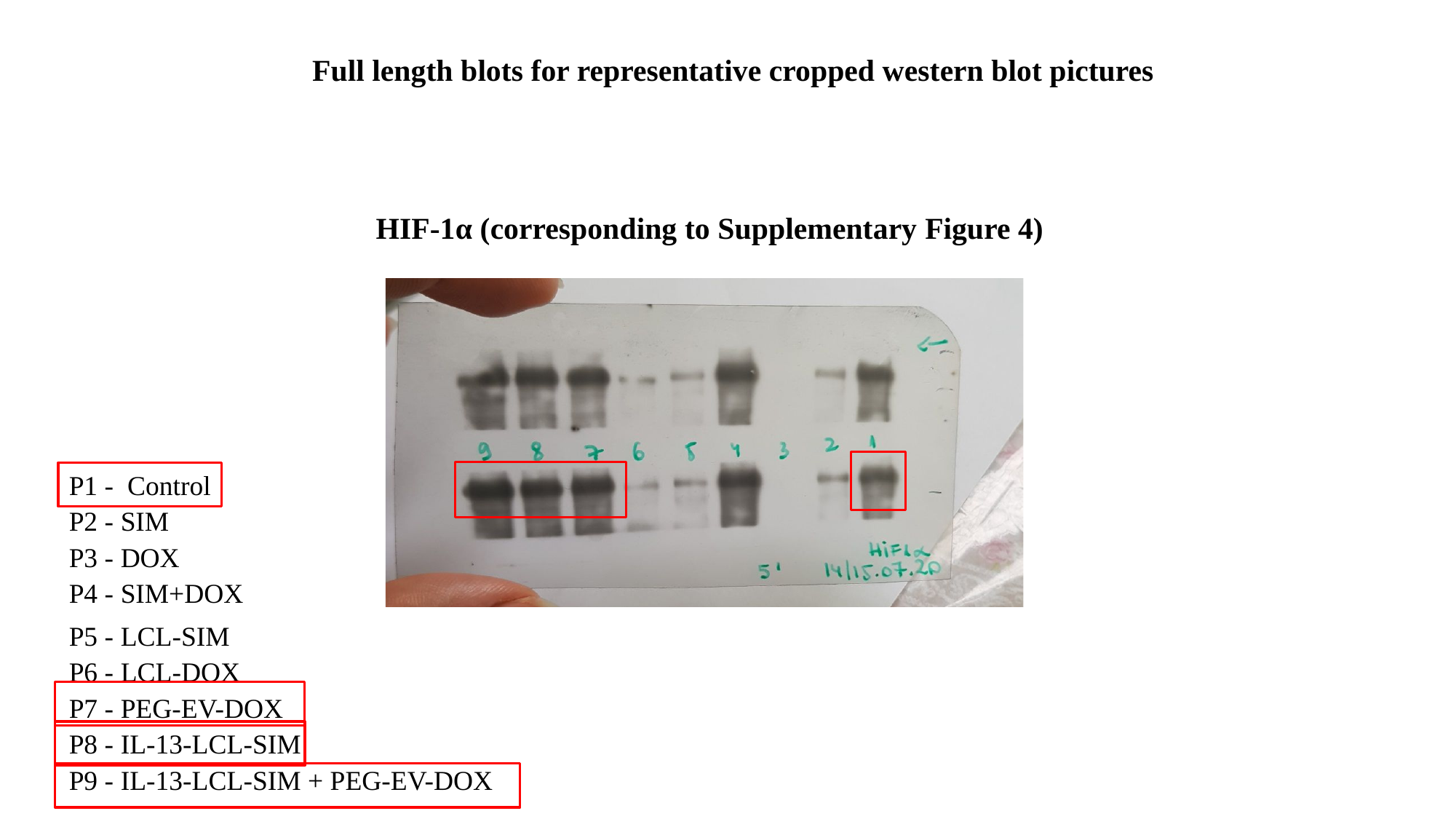

Full length blots for representative cropped western blot pictures
HIF-1α (corresponding to Supplementary Figure 4)
P1 - Control
P2 - SIM
P3 - DOX
P4 - SIM+DOX
P5 - LCL-SIM
P6 - LCL-DOX
P7 - PEG-EV-DOX
P8 - IL-13-LCL-SIM
P9 - IL-13-LCL-SIM + PEG-EV-DOX

## Slide 10
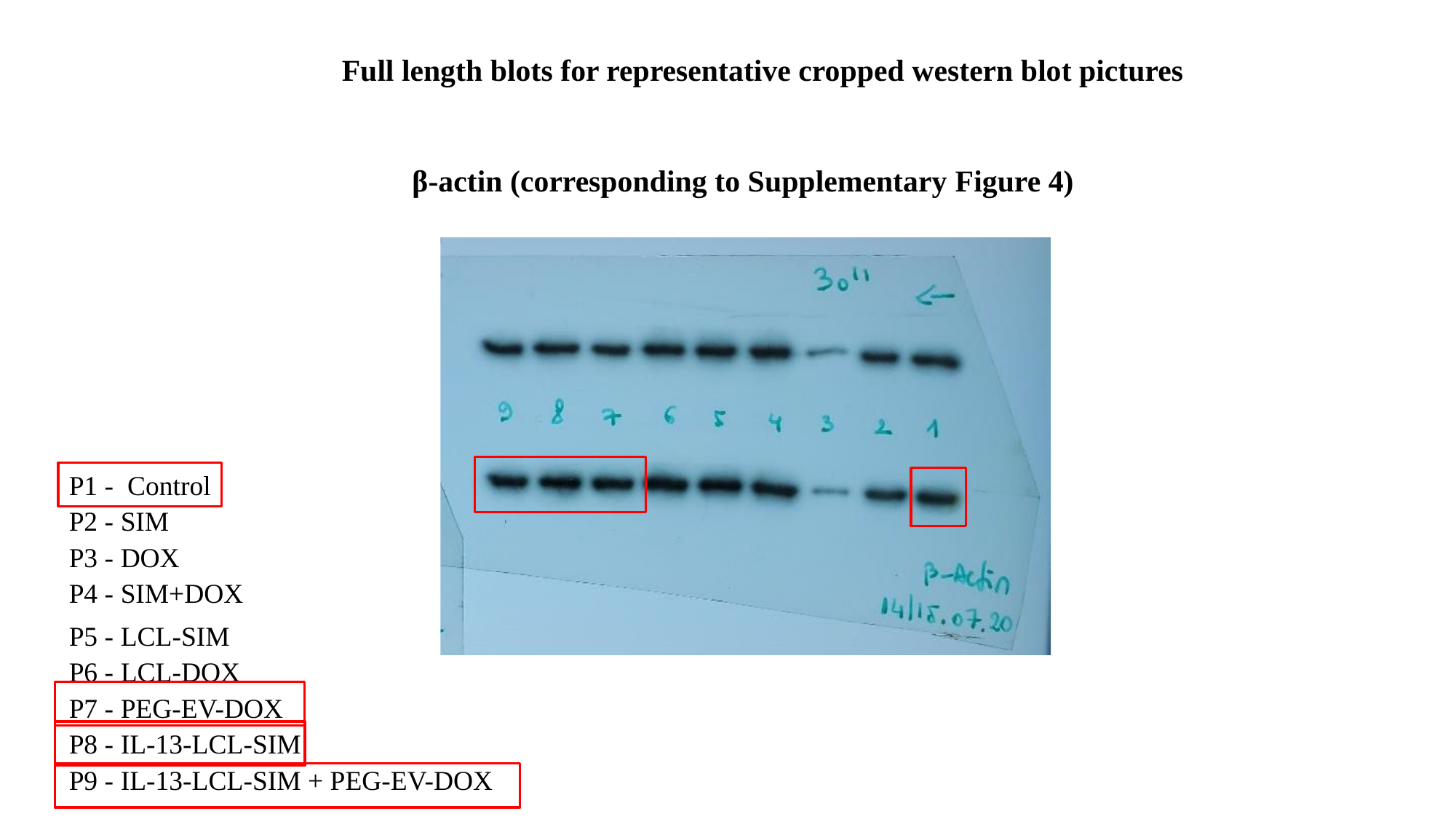

Full length blots for representative cropped western blot pictures
β-actin (corresponding to Supplementary Figure 4)
P1 - Control
P2 - SIM
P3 - DOX
P4 - SIM+DOX
P5 - LCL-SIM
P6 - LCL-DOX
P7 - PEG-EV-DOX
P8 - IL-13-LCL-SIM
P9 - IL-13-LCL-SIM + PEG-EV-DOX
